# Supplementary material for: Improving early prediction of crop yield in Spanish olive groves using satellite imagery and machine learning
Source: PLoS One. 2025 Jan 15;20(1):e0311530. doi: 10.1371/journal.pone.0311530 (PMC11734994; doi:10.1371/journal.pone.0311530)
Supplement: Attachment — Submitted filename: PONE-D-24-04068.pdf [file pone.0311530.s001.pdf]

PLOS ONE

Early season crop yield prediction using temporal data and satellite imagery with machine learning. A new avenue for planning in Spanish olive groves

--Manuscript Draft--

**Manuscript Number:**	PONE-D-24-04068
**Article Type:**	Research Article
**Full Title:**	Early season crop yield prediction using temporal data and satellite imagery with machine learning. A new avenue for planning in Spanish olive groves
**Short Title:**	Early season crop yield prediction using temporal data and satellite imagery with machine learning.
**Corresponding Author:**	M. Isabel Ramos<br>University of Jaen: Universidad de Jaen<br>Jaén, Jaén SPAIN
**Keywords:**	olive crop; predictive modeling; regression algorithms; multi-source data; Google Earth Engine
**Abstract:**	In the production sector, the usefulness of predictive systems as a tool for management and decision-making is well known. In the agricultural sector, a correct economic balance of the farm depends on making the right decisions. For this purpose, having information in advance on crop yields is an extraordinary help. Numerous predictive models infer accurate crop yield data from aerobiological variables and pollen analysis; this is around spring, in the middle stage of the crop year, when planning and investments are already done. The aim of this study is to anticipate accurate crop yield data at an early stage of the cropping season. In the case of olive groves in Spain, this period is on February. This work is developed for an entire province, Jaen, belonging to the region of Andalusia, in Southern Spain. The methodology uses Machine Learning algorithms together with an exhaustive analysis of predictor variables. Temporal data come from public web services, such as spatial data infrastructures of some state agencies. The processing of the satellite imagery is carried out by the geospatial processing service Google Earth Engine. The result is the early prediction of kilograms of both olive crop and olive oil, with an average absolute error of around 26%. The relevance of this work is the early availability of predicted crop yield data together with the multi-scale applicability of the predictive models. This makes this model<br>a useful tool for all the agents involved in olive grove management. From farmers, to agricultural technicians, researchers and scientists dedicated to the study of the olive tree, to governmental institutions and agricultural associations<br>that provide technical support, advice and regulation to ensure responsible practices and the long-term viability of the olive industry.
**Order of Authors:**	M. Isabel Ramos
	Juan J Cubillas
	Ruth M Córdoba, Master’s Degree student in Geomatic Engineering a
	Lidia M Ortega
**Additional Information:**	
**Question**	**Response**
**Financial Disclosure**<br><br>Enter a financial disclosure statement that describes the sources of funding for the work included in this submission. Review the submission guidelines for detailed requirements. View published research articles from *PLOS ONE* for specific	The author(s) received no specific funding for this work.

examples.<br><br>This statement is required for submission and **will appear in the published article** if the submission is accepted. Please make sure it is accurate.<br><br>**Funded studies**<br>Enter a statement with the following details:<br>• Initials of the authors who received each award<br>• Grant numbers awarded to each author<br>• The full name of each funder<br>• URL of each funder website<br>• Did the sponsors or funders play any role in the study design, data collection and analysis, decision to publish, or preparation of the manuscript?<br><br>Did you receive funding for this work?	
**Competing Interests**<br><br>Use the instructions below to enter a competing interest statement for this submission. On behalf of all authors, disclose any competing interests that could be perceived to bias this work—acknowledging all financial support and any other relevant financial or non-financial competing interests.<br><br>This statement is required for submission and **will appear in the published article** if the submission is accepted. Please make sure it is accurate and that any funding sources listed in your Funding Information later in the submission form are also declared in your Financial Disclosure statement.<br><br>View published research articles from *PLOS ONE* for specific examples.	The authors have declared that no competing interests exist.

Powered by Editorial Manager® and ProduXion Manager® from Aries Systems Corporation

**NO authors have competing interests**<br><br>Enter: *The authors have declared that no competing interests exist.*<br><br>**Authors with competing interests**<br><br>Enter competing interest details beginning with this statement:<br><br>*I have read the journal's policy and the authors of this manuscript have the following competing interests: [insert competing interests here]*<br><br>* typeset	
**Ethics Statement**<br><br>Enter an ethics statement for this submission. This statement is required if the study involved:<br><br>• Human participants<br>• Human specimens or tissue<br>• Vertebrate animals or cephalopods<br>• Vertebrate embryos or tissues<br>• Field research<br><br>Write "N/A" if the submission does not require an ethics statement.<br><br>General guidance is provided below. Consult the submission guidelines for detailed instructions. **Make sure that all information entered here is included in the Methods section of the manuscript.**	N/A

Powered by Editorial Manager® and ProduXion Manager® from Aries Systems Corporation

**Format for specific study types**<br><br>**Human Subject Research (involving human participants and/or tissue)**<br>• Give the name of the institutional review board or ethics committee that approved the study<br>• Include the approval number and/or a statement indicating approval of this research<br>• Indicate the form of consent obtained (written/oral) or the reason that consent was not obtained (e.g. the data were analyzed anonymously)<br><br>**Animal Research (involving vertebrate animals, embryos or tissues)**<br>• Provide the name of the Institutional Animal Care and Use Committee (IACUC) or other relevant ethics board that reviewed the study protocol, and indicate whether they approved this research or granted a formal waiver of ethical approval<br>• Include an approval number if one was obtained<br>• If the study involved *non-human primates*, add *additional details* about animal welfare and steps taken to ameliorate suffering<br>• If anesthesia, euthanasia, or any kind of animal sacrifice is part of the study, include briefly which substances and/or methods were applied<br><br>**Field Research**<br><br>Include the following details if this study involves the collection of plant, animal, or other materials from a natural setting:<br>• Field permit number<br>• Name of the institution or relevant body that granted permission	
**Data Availability**<br><br>Authors are required to make all data underlying the findings described fully available, without restriction, and from the time of publication. PLOS allows rare exceptions to address legal and ethical concerns. See the PLOS Data Policy and FAQ for detailed information.	Yes - all data are fully available without restriction

A Data Availability Statement describing where the data can be found is required at submission. Your answers to this question constitute the Data Availability Statement and **will be published in the article**, if accepted.<br><br>**Important:** Stating 'data available on request from the author' is not sufficient. If your data are only available upon request, select 'No' for the first question and explain your exceptional situation in the text box.<br><br>Do the authors confirm that all data underlying the findings described in their manuscript are fully available without restriction?	
**Describe where the data may be found in full sentences. If you are copying our sample text, replace any instances of XXX with the appropriate details.**<br><br>• If the data are **held or will be held in a public repository**, include URLs, accession numbers or DOIs. If this information will only be available after acceptance, indicate this by ticking the box below. For example: *All XXX files are available from the XXX database (accession number(s) XXX, XXX.).*<br>• If the data are all contained **within the manuscript and/or Supporting Information files**, enter the following: *All relevant data are within the manuscript and its Supporting Information files.*<br>• If neither of these applies but you are able to provide **details of access elsewhere**, with or without limitations, please do so. For example:<br><br>*Data cannot be shared publicly because of [XXX]. Data are available from the XXX Institutional Data Access / Ethics Committee (contact via XXX) for researchers who meet the criteria for access to confidential data.*<br><br>*The data underlying the results presented in the study are available from (include the name of the third party*	All relevant data are within the manuscript and its Supporting Information files.

and contact information or URL).<br>• This text is appropriate if the data are owned by a third party and authors do not have permission to share the data.<br><br>* typeset	
Additional data availability information:	Tick here if the URLs/accession numbers/DOIs will be available only after acceptance of the manuscript for publication so that we can ensure their inclusion before publication.

Powered by Editorial Manager® and ProduXion Manager® from Aries Systems Corporation

Manuscript Click here to access/download;Manuscript;Ramos et al. 2024.pdf

Early season crop yield prediction using temporal data and satellite imagery with machine learning. A new avenue for planning in Spanish olive groves.

M. Isabel Ramos[a], Juan J. Cubillas[b], Ruth M. Córdoba[c], Lidia M. Ortega[d]

[a]*Department of Cartographic, Geodesic and Photogrammetry Engineering. University of Jaén, Paraje Las Lagunillas, Jaén, 23071, Spain*
[b]*Department of Information and Communication Technologies Applied to Education. International University of La Rioja, Av. de la Paz, 137, Logroño, 26006, Spain*
[c]*Master's Degree student in Geomatic Engineering and Geoinformation. University of Jaén, Paraje Las Lagunillas, Jaén, 23071, Spain*
[d]*Department of Computer Science. University of Jaén, Paraje Las Lagunillas, Jaén, 23071, Spain*

Abstract

In the production sector, the usefulness of predictive systems as a tool for management and decision-making is well known. In the agricultural sector, a correct economic balance of the farm depends on making the right decisions. For this purpose, having information in advance on crop yields is an extraordinary help. Numerous predictive models infer accurate crop yield data from aerobiological variables and pollen analysis; this is around spring, in the middle stage of the crop year, when planning and investments are already done. The aim of this study is to anticipate accurate crop yield data at an early stage of the cropping season. In the case of olive groves in Spain, this period is on February. This work is developed for an entire province, Jaen, belonging to the region of Andalusia, in Southern Spain. The methodology uses Machine Learning algorithms together with an exhaustive analysis of predictor variables. Temporal data come from public web services, such as spatial data infrastructures of some state agencies. The processing of the satellite imagery is carried out by the geospatial processing service Google Earth Engine. The result is the early prediction of kilograms of both olive crop and olive oil, with an average absolute error of around 26%. The relevance of this work is the early availability of predicted crop yield data together with the multi-scale applicability of the predictive models. This makes this model a useful tool for all the agents involved in olive grove management. From

farmers, to agricultural technicians, researchers and scientists dedicated to the study of the olive tree, to governmental institutions and agricultural associations that provide technical support, advice and regulation to ensure responsible practices and the long-term viability of the olive industry.

Keywords: olive crop, predictive modeling, regression algorithms, multi-source data, Google Earth Engine
PACS: 07.05.Bx, *91.62.Bf
2000 MSC: 91D20, 68T05, 62H35

1. Introduction

Agriculture continues to be one of the occupations that contribute most to the economy of many countries in the world. Even though worldwide agriculture impacts in the Gross Domestic Product (GDP) about 6%, crop productivity plays an important role in specific areas, such as the Mediterranean basin. The climate of the countries of the Mediterranean area has conditioned the type of agriculture, focusing mainly on vines, cereals and olives. However, numerous scientific organizations warn that the climate balance is already being affected by climate change. There is growing concern about the lack of rainfall and progressive desertification in the countries of the Mediterranean basin. This fact, together with abnormally high temperatures, has been considered one of the most determining factors for the drop in agricultural production, particularly in the olive sector. The Statistical Office of the European Union (Eurostat) provides data about the olive oil production, indicating a 30% reduction from 2021 to 2022, for the European union as a whole. According to the same organization, Spain, the world's largest olive oil producer (2.5Mha), has seen its production cut in half. The same percentage has been observed in exports. This has had a considerable impact on the price of olive oil, making it three times more expensive in the 2022/23 season than it was three years ago (data from the Spanish ministry of agriculture).

The productivity of crops, including the olive groves (Olea Europaea L.) depends on various factors such as soil fertility, weather condition, the type of tillage performed and also the amount of harvest of the last year. Some of these factors are controllable by the farmer, but others cannot be acted upon directly. Good harvests are a fundamental objective for farmers, but also for oil mills, distribution and insurance companies, as well as the agri-food in-

dustry. Early crop forecasting is very important to know the future behavior of the markets. In the particular case of olive oil production, it allows to establish market strategies, to know in advance the income of farmers and companies, the number of workers to be hired, or the machinery that will be necessary. This information would also allow adjusting production costs in terms of the use of phytosanitary products or tillage.

The importance of early crop prediction is a hot topic in the scientific literature. In the particular case of the olive grove, most of the work focuses on the study of variables such as the amount of pollen in the air, vegetative indices that indicate plant health, or rainfall maps. However, some of these values are captured in springtime between April and May, when there are already other visible features that allow agricultural technicians to predict how abundant the next harvest will be. In this sector, an early forecast is considered in the months of February, just when the harvesting of the previous season has finished. Although not all the factors that will affect the next harvest are known at this stage, it would be advisable to advance what the general trend will be if no other negative factors intervene. In any case, this predictive model will be fed as the seasons progress to better adjust to the time of harvest.

In this work, we focus on the early crop yield prediction based on the most representative variables such as: the previous year's production, rainfall at specific times, other meteorological factors in the previous autumn and winter, as well as the most relevant vegetation indices. The process has been carried out using Machine Learning (ML) techniques, a branch of the Artificial Intelligence (AI) capable of making an early prediction in many research fields. The system has been trained by collecting the values of all these variables over the course of a decade. This early forecasting process has been carried out throughout all the municipalities of the province of Jaén, the area with the highest concentration of olive groves on the planet. The variables to be predicted are both the quantity of kilograms of olives and the quantity of oil obtained. The difference between these two values is known as the harvest yield, and can vary from season to season.

The paper is structured as follows. Section 2 gives an overview of the state of the art in relation to the techniques and data input used in this early prediction study. Section 3 describes the area covered by the study and the characteristics of its olive grove plantations, as well as the workflow of the methodology developed. In Section 4 the results of the different tests carried out are analysed, justifying the reasons for each test and deepening

Year	Tons
2021	1.317.607
2022	2.129.577
2023 (provisonal)	2.064.070

Table 1: Tons of olive oil in Spain

the knowledge offered by the resulting data. Finally, Section 5 includes the main conclusions of this q work and proposes further applications.

2. State of the art

Spain is the largest producer of olive oil in the world, and within this country, the province of Jaén (in the south of the country) is the most productive. This is the main economic activity in the area. According to data published by the Spanish Ministry of Agriculture, Fisheries and Food (MAPA) in 2020, the province of Jaén (2,67% of the national territory) represents almost 24% of the olive grove area in Spain [1]. The most recent data for the current 2022/23 crop year show a significant drop in production compared to the previous years, as depicted in Table 1, in which an important descend in the production is observed. This decline was already been predicted by agricultural technicians and farmers at the end of spring 2022. Since then, olive oil prices have risen steadily, mostly because the majority of forecasts for the next growing season are also negative in terms of production.

This forecast for the next harvest portends an upward trend in olive oil prices. Companies in the sector are studying new market strategies for the coming months. For all the agents involved in the sector, having this prevision at the beginning of the year gives them an advantage in establishing these commercial strategies. Summarizing, this forecast impacts in the economy of farmers and companies as described below:

- **Farmers:** Many families in Jaén and the rest of the Mediterranean basin derive their income from agriculture. An early forecast allows them to adjust their household finances in case of a low forecast, or take new expenses and anticipate the hiring of new personnel in the opposite case.

- **Agricultural cooperatives and oil mills:** In the area under study,

it is common for farmers to form cooperatives. They guarantee the milling process, sale of the product and all kinds of consulting services.

For them or for the oil mills that work as external companies, early forecasting allows them to better adjust the prices at which they market the product. It can help to know in advance the personnel to be hired, or to improve facilities to face a successful campaign.

- **Insurance companies:** These companies move a lot of money and are particularly interested in knowing the risks and adjust premium prices or indemnities in order to establish their rates.
- **The food and distribution industry:** Olive oil is one of the most appreciated oils because of its culinary and healthy properties. These companies have to make decisions about the most advantageous moment to market the oil.
- **The international market:** Spain is the world's largest exporter of olive oil, with 65% of its total sales. Knowing its production in relation to other producing countries allows to establish better market strategies.
- **The agricultural machinery industry sector:** Over the past decade, the olive industry has experienced a significant surge in mechanization. This mechanization primarily targets the reduction of harvesting expenses, which represent the highest cost within olive farming. Early forecasting makes it possible to better adjust machine production to actual demand in advance.

In view of the above, the advance in the prediction process is challenging from an economic as well as a scientific point of view. Crop prediction is key for many growing types. Although, some of them such as olive groves, have a long vegetative cycle with numerous factors affecting pollination and fruit set. Various factors, including climatic conditions during different growth stages, disease prevalence, olive tree species, and the previous year's yield, are recognized to influence the outcome of the next harvest. Scientists and farmers strive to identify these and numerous other variables to better understand their impact on crop production.

Agriculture has undergone many changes from its beginnings. Initially due to the mechanization of crops and agrochemical use, and more recently

due to digitization and the incursion of ICTs (Information and Communication Technologies). In the past, crop predictions heavily relied on farmers' expertise, but today, much of agricultural research centers on improving crop forecasting. This shift has been complemented by other economic sectors using the predictive potential of Artificial Intelligence (AI) techniques, specifically Machine Learning (ML) and Deep Learning (DL). These techniques are now extensively employed for crop yield prediction [2, 3, 4, 5]. This helps the farmer to make decisions for the best management strategies.

DL or ML regression techniques are mostly used for determining the degree to which independent variables influence dependent variables. In the particular case of the olive grove, it is possible to establish a relationship between crop production (dependent variable) and other previously referred variables, such as vegetation indices, temperatures or irrigation [6, 7], plant health [8], the previous harvest, even the terrain slope or the age of the olive tree [9] (the independent variables). There is undoubtedly a correlation between these factors. Years of abundant harvest are typically marked by well-distributed rainfall throughout the fruit development stages, adequate temperatures and a moderate yield form the previous year. Production is obviously also related to the agricultural practices employed by farmers, encompassing activities such as tree pruning, the application of fertilizers, and the use of various phytosanitary products aimed at managing pests and weeds.

In this sense, most works related with crop prediction applies regression techniques to some of these factors independently or through a combination of them. One of the most studied variables is the amount of pollen in the air [10, 11, 12]. Although there is a direct relationship between pollen quantity and harvest, this factor alone is not decisive, as it also affects other factors such as water deficit, extreme temperatures or phytopathological problems between flowering and harvest [13]. The most reliable prediction occurs once fruit set has begun, what happens after pollination is complete. Going further, high crop yields usually imply high oil production, although with certain nuances.

However, all these examples of crop yield forecasting are considered *late prediction*, since the information is provided only five months before the next harvest [12, 14]. This information is collected when the tree is already in bloom, whereas our objective is to make the prediction two months earlier, on February. Another type of prediction focuses on oil production. In [15] it is introduced a conceptual model for predicting fruit oil content in order to establish the optimal harvesting period, but it takes place at even later times.

In fact, only a few papers are really focused on early estimation in the literature [16], but some of them actually considering the estimate in spring months instead of the winter prior to harvesting [17]. Therefore, in this paper we first study the influence of the most representative variables in early crop prediction according to the related bibliography and the agricultural technicians consulted: vegetation indices, climatic conditions and the previous harvest information.

2.1. Spectral information and vegetation index

Remote sensing applications provide a variety of information on all types of crops. In the last years, an increasing source of information is provided by unmanned aerial vehicle (UAVs) platforms, mainly equipped with optical multispectral cameras, to map, monitor, and analyze, temporal and spatial variations of vegetation using spectral vegetation indices (VIs). However, for this stydy, historical data series were not available from this source.

Satellite imagery is also widely used despite spatial resolution is not adequate to capture the surface variability required in many precision agricultural systems. However, they are increasingly improving temporal and spatial resolution, being sufficiently precise to carry out studies at municipality level, as is the case in this work. The advantages are accentuated by their accessibility, larger area coverage, less processing and operator interaction coupled with more spectral information. In any case, satellites and airborne multispectral cameras are not opposing technologies, they can be used together as complementary data.

Multispectral and hyperspectral sensors generate images consisting of multiple bands wavelengths. Linear combinations of them, the so-called vegetation indices, are aimed at highlighting relevant features about plants and soil. In particular, a total of 82 spectral vegetation indices were collected from the literature by Pu, Ruiliang [18]. Between this index set, the most widely used is the Normalized Difference vegetation Index (NDVI), whose relationship with olive grove production has already been reported [19]. Literature has studied the relationship of this index and the use of fertilizer in olive groves [20, 21], water deficit and antioxidative enzyme activity [22]. The fact that the index behaves differently depending on the type of vegetation, has served to differentiate each of the studied crops [23].

In addition to the NDVI index, some studies work with a combination of some others to determine which of them perform better for different crops [24, 25]. For instance, in [26] a set of eleven spectral values are analyzed

Index	Index name	Formula	Bibliography
NDVI	Normalized difference vegetation index	$\frac{NIR-Red}{NIR+Red}$	Used in most papers
GNDVI	Green normalized difference veg. ind.	$\frac{NIR-Green}{NIR+Green}$	[25],[26],[27],[21],[8]
MSI	Moisture Stress Index	$\frac{NIR}{SWIR}$	[29, 30, 31]
NDMI	Normalized Difference Moisture Index	$\frac{NIR-SWIR}{NIR+SWIR}$	-
NDRE	Normalized difference vegetation index	$\frac{NIR-RedEdge}{NIR+RedEdge}$	[27],[28],[8], [26]
GRVI	Green Red Vegetation Index	$\frac{Green-Red}{Green+Red}$	[7]
GCI	Green Chlorophyll Index	$\frac{NIR}{Green} - 1$	[25]
SAVI	Soil Adjusted Vegetation Index	$\frac{NIR-Red}{NIR+Red+L} * (1+L)$ $0 <= L <= 1$	[27],[28],[8], [26]

Table 2: List of vegetation indices included in the literature related to olive cultivation.

to determine the density of vegetation ground cover. Table 2 provides information about a selection of these and other indices in the scope of olive grove studies. It has been reviewed which vegetation indices are related to vegetation vigor in [8, 27, 28], including indices such as NDRE or SAVI between others. Water stress has been related to GRVI, MSI and NDMI indices [7, 29, 30, 31], fertilization with the GNDVI [21] or vegetation density [26] using GCI. On the contrary, some well known moisture-related indices such as the Normalized Difference Moisture Index (NDMI), have not been related so far to crop prediction in olive groves.

As the first step in our methodology, these indices included in the bibliography have been checked in order to determine their relationship with crop prediction. Some of them have been discarded, and on the contrary, some others have been added to determine which of them most contribute for forecasting in the particular olive grove case.

2.2. Meteorological variables

Crop yields depends to a great extent in climatic conditions. As crop conditions are being affected by climate change, some studies are being conducted to study the viability of long-term crops in relation with the geographic location [32].

The olive grove is perfectly adapted to the Mediterranean climate and the plant usually survives adverse meteorological situations. However, production is determined by rainfall (or irrigation) and temperatures [33, 34, 35]. In

fact, low rainfall conditions and high temperatures are considered to be the most important factors influencing the production decline for the 2022/23 and 2023/24 seasons in Spain. According to climate reports, the rainfall pattern has changed in the last seven years. Long periods of drought contrast with torrential rains in a short period of time, with little capacity to infiltrate the soil or supply the reservoirs.

ML and DL Regression algorithms are traditionally used to correlate meteorological data with the harvests obtained. The capture of this information can be performed both using meteorological stations or satellite data. In [36], the use of satellite data from Copernicus' ERA5 is advocated for modeling phenological phases of olive orchards, since these models provide better predictions in comparison with those trained with weather station temperature measurements.

Along with others, many research works use weather variables in regression algorithms for the olive grove sector [12, 37]. Differentiating between climate variations by seasons is significant since it affects the different vegetative stages of the plant and the fruit [14]. In [38] this influence is related to the presence of the olive fruit fly, which is one of the pests that most affects this crop. In any case, rainfall is also a relevant factor in areas with shortages, such as the Andalusia region in southern Spain. In fact, drought effects on vegetative health and its effect is detected by analyzing vegetation indices, such as NDVI, GRVI [7], NDWI (Normalized Difference Water Index), NDMI (Normalized Difference Moisture Index), MSI or SAVI. [29, 3].

Drought problems occurs even in irrigated crops. Support irrigation of olive trees consists of providing water at specific moments in order to save the crop. 57% of the Andalusian olive grove area (south of Spain) is irrigated, in contrast to the Spanish national average of 22%. Irrigation makes it possible to mitigate water shortages at critical moments, such as flowering, fruit set and stone hardening (the first stage in the biological formation of the oil in olive fruits). In these cases, a water deficit would cause the fruit to fall. In any case, water shortage affects the reservoirs, and that leads to sparse irrigations aimed at saving the plant, rather than the crop. Olive groves need at least 0.4 m^3 per square meter per year. Irrigation contributes to guarantee water supply, but this supplement may not be sufficient since additional factors are also involved. Water status monitoring is crucial to establish an automatic schedule irrigation, so that plants are not overwatered or suffer a water deficit that limits production.

Water stress can also be detected by specific vegetation indices [7, 24, 30].

This analysis is usually focused on in the spectral response of the soil with indices such as NDMI (Normalized Difference Moisture Index) or SAVI (Soil Adjusted Vegetation Index), this last used to detect the influence of soil brightness in areas where vegetative cover is low.

Hyperspectral imaging surpasses multispectral cameras in resolution by obtaining a much larger number of spectral bands. This technology can anticipate drought stress by analyzing enzyme activity, photosynthetic rates [22] or the leaf water potentials by analysing specific vegetation indices [29]. In any case, climate is not the only variable to be considered, as other favorable conditions for a good harvest have to be considered and correlated, as it is the plant's health [39]. On the other hand, some pathogens such as the bacterium Xylella fastidiosa restrict water and nutrient flow through xylem, producing a similar set of symptoms that can also be mistaken with water stress [40].

Despite all the research work, only a few studies focus on determining which set of combined factors are really relevant for this predictive study. In [41, 42] climatic variables along time and VIs are jointly used for crop forecasting. Some recent works obtain the information about meteorological variables, such as soil moisture, by analyzing specific vegetation indices [30]. It is common to face an inherent problem of merging data from different sources, considering variable accuracy and scale levels between them [25].

Summarizing the section, Machine Learning (ML) can determine patterns and correlations and discover knowledge from datasets. In a fist phase, the model is trained with historical data, allowing to properly determine this model, since the results are obtained from past experience. In the testing phase, part of the historical dataset is used for training, and the rest for evaluating performance. Crop yield prediction is one of the most challenging problems in precision agriculture, specially in early prediction, when the plant does not yet show appreciable signs of flowering. In the case of olive orchards, an early prediction refers to the month of February, just before the beginning of spring.

The aim of this work is to model an early prediction of olive crop yield at the municipality level using regression analysis algorithms. The olive crop yield is understood as two variables: on the one hand, the number of kilograms of olives to be harvested, and on the other hand, the amount of olive oil obtained. It is necessary to understand that both variables are related but not in direct proportionality. Harvesting a large number of Kg of olives does not lead to obtain a large number of Kg of oil, and vice versa. This

predictive work requires the use of several variables, as crop yields depend on many different factors, so it is essential to adapt the study not only to the crop under study, but also to the specific characteristics of the crop and its geographical location.

3. Material and Methods

3.1. Study area

The study area comprises all the municipalities of the province of Jaén, extending from 38°N 3°W (ETRS89) in Andalusia, southern Spain. The province of Jaén spans a total area of 13496 km^2, encompassing diverse topographies ranging from mountainous regions to extensive valleys, with a total of 97 municipalities (see Figure 1.a). The climate is Mediterranean, partially varied according to the orography and the proximity to the Guadalquivir river that crosses the whole province. The average annual temperature is 15-17 °C with a wide temperature range between day and night, especially in those municipalities close to the river. Rainfall is around 500 mm and accumulates in the coldest period, although this factor varies according to the orography of the area.

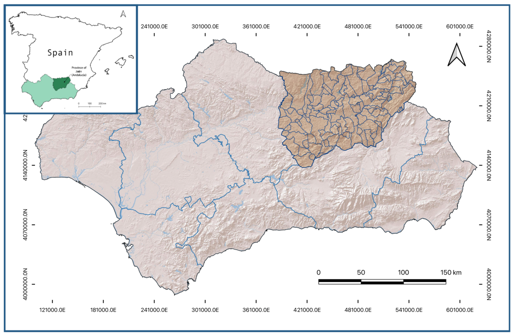

(a) Jaén location.

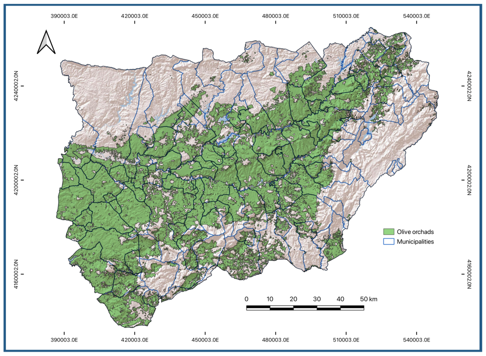

(b) Land occupied by olive grove in Jaén.

Figure 1: (a) Location of our area of research in Andalusia, Southern Spain. (b) Land uses for olive grove in the province of Jaén. The coordinates (m) are UTM, zone 30, referred to ETRS89.

Knowing these characteristics, the data obtained including rainfall, temperature and vegetation index values are associated with each of the municipalities. This information is aggregated by seasons and municipalities to obtain averages from each one.

Although a significant portion of the province of Jaén is dedicated to olive cultivation, the extent varies depending on the municipality and the specific orographic zone within the province. Some municipalities are located in mountainous areas where olive orchards are scarce, while others have diverse land uses. Additionally there are population centers, reservoirs, ponds, rivers, and road networks mottling the province. This requires to distinguish among the different land uses in order to identify those devoted to olive crop. Thus, all calculations are made using data focused on these selected areas, avoiding the rest of the area of the municipalities without olive cultivation. Otherwise, the information related to these areas would insert noise in the dataset.

This information is extracted from SIGPAC[1], a Geographical Information System for the Identification of Agricultural Parcels, which is maintained by the competent agricultural Delegation of the Andalusian Government. SIGPAC contains updated information until 2023.

On Figure 1.b), it is noticeable the amount of land occupied by this type of cultivation, as well as the administrative division of municipalities. As said, some of them have a vast amount of olive cultivation while others barely have some plots. Furthermore, several production classes of olive trees are recognised, in relation to the average yields of each plant or the cost of cultivation. In this sense, it is worth noting the great difference between olive groves located in the mountains, which occupy marginal soils, and modern high-intensity plantations located in areas with more favorable agronomic characteristics. Between the two, average yields can vary by up to 30 times the total volume (500 kg/ha/year versus 15000 kg/ha/year) [43].

—The selection of the different land uses has been performed with QGIS[2] software, in which only those plots of olive trees have been considered. Later, this vector information is uploaded to Google Earth Engine to process the spectral images, and extract only the information related to these areas. In Figure 2, it is possible to observe the simplification process from the first data obtained. This tool allows to perform massive calculations in an agile way, and as the study is done at the scale of a municipality, small details are not compromised.

[1] https://www.juntadeandalucia.es/organismos/agriculturapescaaguaydesarrollorural/servicios/sigpac.html

[2] https://www.qgis.org/es/site/

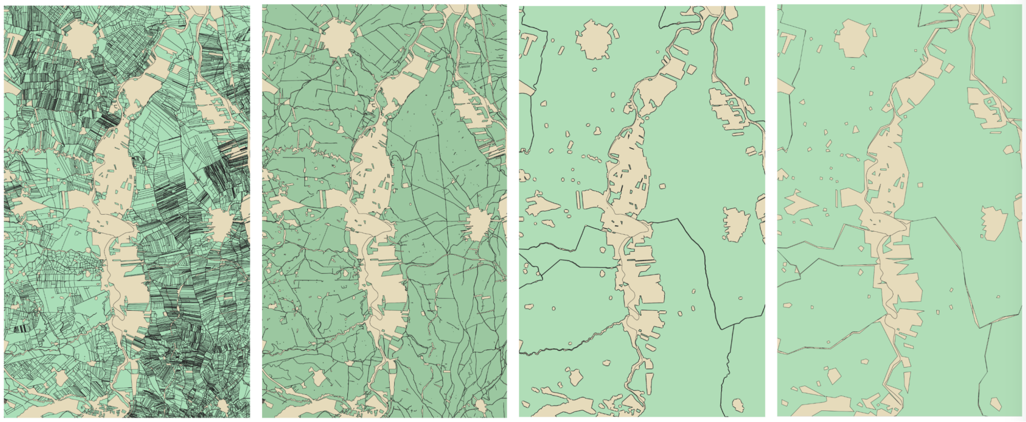

Figure 2: Vectorial simplification process.

3.2. Dataset

The fundamental basis of any predictive study is the availability of reliable data to ensure a quality prediction. In this case study, the dataset consists of information from official sources and agencies, guaranteeing data reliability. This accuracy in the results sometimes involves the need for some pre-processing such as, outlier removal, data cleaning, classification, data normalization, etc.

The olive tree has a vegetative cycle of two growth stages in which the care and feeding of the plants is essential to guarantee a good yield after harvesting. Different factors influence the cultivation of the olive tree, some of which are specific to the place where they grow, such as environmental and meteorological factors. Others, however, are a direct consequence of the farmer's agricultural practices on the farm. Thus, within these two stages, the olive trees go through a phase of budding (February-April), flowering (April-May), fertilization and fruit set (May-June), fruit growth (June-September), fruit ripening (October-December) and vegetative rest (November-February). In this work, these stages are grouped into three, namely seasons, which coincide with the times of the year when the olive tree responds uniformly to the external factors acting on it. Table 3 shows the months that include each season considered.

In general terms, the yield of the olive grove depends on the climate, the quality of the soil, the presence of pests, tillage, olive tree varieties and planting density. However, this study is conducted at municipal aggregation level, where several of these factors are inherently embedded in the harvest data

Year	Dates
Winter	01-December to 29-February
Spring	01-March to 30 - June
Summer - Autumn	01-July to 30-November

Table 3: Season division.

or the municipality's geographical context, making it unnecessary to explicitly insert them into the dataset. For the research, influential data that do not conform to a recognizable yearly pattern are employed as training data. Therefore, environmental and meteorological variables are considered, as well as variables that indicated the state of the crop during the crucial seasons. These are certain vegetation indices that are calculated from satellite images. In fact, the influence of data differs depending on the two objectives of the study, the kilograms of olive crop or the kilograms of olive oil. Furthermore, the impact of the same data on the predicted target may vary based on their values at a particular date.

3.2.1. Olive crop yield data

The Regional Government of Andalusia publishes annual olive crop yield data at different levels of aggregation. In this work, as mentioned in previous sections, data grouped at a municipal level of detail are used. The research utilized crop yield data from every municipality in Jaén, encompassing a dataset spanning thirteen years, from 2009 to 2022. Crop yield is understood as a double variable, on the one hand as the quantity of kilograms of olives harvested annually, and on the other hand, as the kilograms of oil extracted. In this sense, during the harvesting period, the farmers take the harvested olives to the mill every day. There, the harvest is poured into a weighing system, which is periodically checked on the basis of metrological systems regulated by governmental law, thus complying with the current ISO 9001 and ISO 17025 standards. Once olives are in the mill, the olive oil extraction process begins immediately. In this step, the whole olive fruit is crushed by means of a cold mechanical process. Afterwards, the mass undergoes a cleaning and centrifuging phase to separate the oil from the rest of the impurities. The quantity of oil obtained varies depending on the quality of the olives harvested. Not all olive harvests produce the same amount of olive oil.

Even though the two targets of this research (Kg of harvested olives and

Kg of oil) have a certain relationship of dependence, the dataset influences each one in a different way. For this reason, a corresponding predictive model is generated for each one.

3.2.2. Weather information

Despite having access to meteorological station data scattered throughout the province of Jaén, both temperature and precipitation, they do not cover all municipalities within the province. This implies the need of data interpolation, resulting in values that deviate from reality on the one hand, and areas with no information available on the other. For this reason, we decided to use satellite data, as it provides homogeneous information for all affected regions. As previously explained, the months are grouped into seasons according to the olive tree necessities. Satellite data allows to select precisely the dates of interest for this analysis.

In the case of temperature data, the focus is on the warm months, namely May and June. During this time period, maximum temperatures is analysed, as they play a critical role in determining the health of the olive blossom, especially when they rise too high. This period represents the tree's greatest vulnerability with respect to that year's crop, as it is directly dependent on the flowering process. The productivity of the crop will vary according to the state of the flowers during this period. During the rest of the year, temperature is not as critical. Considering that this study is an early, pre-flowering prediction, it is expected that the temperature variable would have little weight in the model. Nevertheless, its influence is checked, although the main focus is mainly on humidity and rainfall patterns.

Conversely, when considering precipitation data, calculations are necessary throughout the entire year. The total precipitation for each season is determined by summing the values, as opposed to calculating the average, as done with other variables.

The meteorological data is incorporated into the model forming labels rather than numerical values, reducing noise within the dataset. Using labels instead of numerical ranges leads to more data repetition than if numerical values are used. Furthermore, this approach normalizes all available information, creating a more straightforward system. Therefore, specific ranges are defined for both temperature and precipitation. Based on these ranges, labels are generated, such as 'low-medium-high', depending on the values obtained for each municipality, which reduces noise in the model.

Satellite	Plataform	Spatial Resolution	Revisiting Time	Operating Timeline
Landsat 7	USGG	30 m	16 days	1999 - present
Landsat 8	USGG	30 m	16 days	2013 - present

Table 4: Landsat 7 and 8 characteristics.

3.2.3. Satellite data

Taking into account the information to be obtained, the choice of satellite type becomes a critical decision. Consequently, different satellites are used to acquire data related to different fields, as it is vegetation indices, temperature or precipitations.

- **Vegetation Index:** In the context of vegetation index calculations, the study is initially based on Sentinel-2 imagery, since it has complete information with good resolutions available to the public, and possesses a wide spectral capacity. However, the available crop yield data spans from 2009 to 2021 rendering it impractical to solely employ Sentinel-2 for vegetation index computations. This is due to the fact that this satellite has been operational since June 2015, thus resulting in a data gap prior to its launch. As an alternative, the next best option are the Landsat satellites, since they have similar spatial and temporal resolution as Sentinel, if not slightly poorer, and with enough multispectral capacity to do the corresponding calculations. Landsat has launched nine satellites up to use for the public, all on different dates. For this case, since the data goes back to 2009, we finally use Landsat 7 and 8, combining their information since 2013 along with the launch of Landsat 8. The more images there are per season, the more reliable is the data obtained from them, given that some images are discarded after the filtering process of cloud masking.

  With the use of Landsat 7 and 8, all the vegetation data possible are obtained, counting with the limitations of said satellites, such as their spatial and temporal resolution. Table 4 summarizes the general characteristics of such satellites.

  The most pressing issue, given the division of seasons, is the presence of clouds, which disables the possibility of carrying out the calculations correctly. This problem is solved by increasing the number of images of the same area, this way the pixels that contain clouds can be eliminated

and still are supplied by others which do have the information. The more images available, the more reliable the calculated indices can be, since it portrays more accurately the land's reality. With this mindset, it is decided to combine the imagery of both satellites.

Calculations are made with data from 2009. However, until June 2013, the available images are only from Landsat 7, which causes lulls in some seasons, specially in rainy winters. As a consequence, it is not always possible to obtain images without clouds. To clarify, the winter of 2010, as well as 2011, is not adequately processed due to the absence of cloud-free images for certain municipalities. At a model level, this provokes the discarding of the affected years and the previous to these, since the algorithm operates by taking the current year of the observation and the previous one. Therefore, the model starts properly operating from 2012.

As it is specified beforehand, several indexes are picked to be applied into this field of study, each one with specific characteristics and different utilities. This initial collection, as shown in Table 5, is drawn from scientific papers and various sources that focus on predicting crop outcomes and conducting surveys. For this work, it is preferable to have information about leaf chlorophyll, soil moisture in addition to vegetation stress and health.

It is important to have in mind the characteristics and limitations of this data, for instance, the satellites resolution. In this particular case Landsat 7 and 8, where the majority of bands have a resolution of 30 meters.

Typically, olive orchards do not have dense vegetation due to significant spacing between each plant. Therefore, it is important to consider that the area studied tends to have a predominance of soil exposure. Likewise, the study is centered on cultivated land rather than aquatic areas, so it is advisable to select moisture indices over others that focus solely on that kind of surface.

After conducting an analysis of each index within the study area, and after performing various comparative assessments among them, certain indices are excluded from further consideration. The criteria for exclusion are based on their practical utility, the satellite sensor's capability to calculate them accurately, or the presence of redundant information.

Index	Name
NDVI	Normalized Difference Vegetation Index
EVI	Enhanced Vegetation Index
SAVI	Soil Adjusted Vegetation Index
CGI	Green Chlorophyll Index
ARVI	Atmospheric Leaf Index
GLI	Green Leaf Index
RGR	Red Green Ratio
SIPI	Structure Insensitive Pigment Index
NBRI	Normalized Burn Ratio Index
NDRE	Normalized Difference Red Edge
GNDVI	Green Normalized Difference Index
MSAVI	Modified Soil Adjusted Vegetation Index
NDWI	Normalized Difference Water Index
NDMI	Normalized Difference Moisture Index

Table 5: Main vegetation indices.

When feeding the predictive model, it is more efficient to provide simple, concise and precise information. Hence, it is advisable to avoid inputting redundant data, as it would introduce noise to the model.

Table 6 summarizes all the indices that have been discarded and the reasons behind this decision.

**Index**	**Characteristics**
MSAVI	The primary distinction between this index and SAVI lies in the manner in which they are adjusted to account for the effect of bare soil, thereby yielding comparable values. Given the type of terrain in which olive cultivation occurs, MSAVI would be a more suitable index since it mitigates the noise influenced by bare soil, enhancing data quality. However, the calculation of this index requires a multispectral band classified as 'red-edge', which is not available in Landsat 7 and 8. Therefore, the MSAVI index is excluded from consideration, using SAVI index instead.

RGR	This index allows the calculation of relative expression of lead reddening caused by anthocyanin in relation to chlorophyll. Given that the study pertains to olive trees, which maintain leaf color throughout the year, this index is not deemed essential for the investigation. For the assessment of chlorophyll content and the overall health or vegetation stress, there are more specific indices available.
NBRI	This index is primarily employed for the identification and visualization of areas affected by wildfires, therefore, it is not aligned with the focus of this study. In the case of analyzing drought in a specific area, it is more reliable data obtained by the NDMI or other vegetation indices. Given the specific selection of olive orchards as the study area, it would be unusual to find a significant number of them affected by wildfire.
EVI	This index represents an enhanced tool for quantifying vegetation health over areas characterized by high canopy density. However, as with the GNDVI index, the study area lacks dense vegetation; in fact, the opposite condition prevails. Upon analyzing the data obtained through this index calculation, a significant disparity in the results is appreciated, which could indicate inadequate application or methodology. Consequently, it is advisable to exclude this index from consideration within the scope of this study.

GNDVI	This index is responsible for calculating the vegetation health and greenness, in a similar connotation that NDVI, but with a higher saturation point. It is designed for its use in crops with dense canopies or in more advanced stages of development. However, in the case of olive orchards, these conditions of dense vegetation are not typically present. In fact, with the use of other indices like CGI, it is possible to obtain similar classifications with even more pronounced contrast, so again, GNDVI index is considered redundant.
NDRE	Similar to the MSAVI index, Landsat 7 and 8 satellites lack the necessary multispectral 'red-edge' band required for the computation of this index. For this reason, and given that we already have an adequate array of indices for monitoring vegetation status, it is excluded from the study.
NDWI	The utility of this index is the detection of water presence over land, typically used for direct monitoring of aquatic areas, such as tracking lake droughts or floods. With this insight, it does not make sense to apply it in this study, since the track of water flow is not needed. In contrast, with the NDMI index, it is possible to assess both vegetation and soil moisture, being useful for comparisons related to droughts or crop health.
MSI	With this index it is possible to assess vegetation water stress, using both infrared and thermal bands for said purpose. While NDMI evaluates moisture in both vegetation and soil, MSI is primarily focused on detecting plant water stress by measuring vegetation temperature. The purpose of applying MSI and the index SIPI are quite similar, since both detect vegetation stress. Moreover, the results obtained from MSI and NDMI are practically equal so it is advisable to dispose of one of them.

Table 6: Vegetation indices selection.

- **Temperature:** This is monitored using MODIS constellation, given that it has spectral bands that allow the detection of surface temperature. Again, this is not the data with more resolution available, but is the most suitable one that has information since 2009. Despite its less refined spatial resolution, calculating surface temperature using this satellite ensures consistent information across the years, presenting an advantageous feature. MODIS provides two temperature values, one from the day where it is extracted the maximum, and another from the night where the minimum is selected. This way, it is possible to consider both ranges in a way that accommodated more with reality. It is also feasible the use of Landsat satellites for this purpose, but the data obtained do not represent the reality of the maximum and minimum temperatures of the province, since the images are always taken around the same time of the day.

- **Rainfall:** For rainfall information, we use ECMWF data, based on Copernicus satellite images and Google information. Specifically, ERAS5 (Earth Climate Reanalysis) project, which uses numerical models and observations to create a complete dataset on a global scale. It is not an image collection per se as the other cases, but is processed as one, obtaining data on millimeters of depth. As the data are processed by seasons that compile different numbers of months, the information obtained is the sum of rainfall in this time period.

All these values are obtained by means of Google Earth Engine, a platform that is able to collect all this satellite imagery without the need of the user to download them, allowing remote processing. This platform combines an extensive catalog of satellite imagery and geospatial datasets with analysis capabilities at different scales. This approach enhances processing speed as data downloading is only required after final calculations in the form of numerical tables. This way, there is no need to download the large amount of information associated with satellite images.

3.3. Modeling methodology

The methodology followed consists of the following phases, from the understanding of the data, its acquisition and preparation process, the gener-

ation of the models and, finally, the validation or analysis of the accuracy obtained. The output data of the model is twofold: the amount of olive harvest (the number of Kg of olives) and the amount of oil, which depends on the yield of the fruit. This information is collected for each campaign and for each one of all the province municipalities. In this approach, the study is based on a supervised machine learning analysis, where the value of an unknown variable (crop yield) is deduced from a few known variables.

The workflow of the methodology carried out consists of algorithms and data mining techniques used as follows in each phase:

1. *Data extraction and loading.* Harvest and olive oil datasets are public information provided by the regional governmental institution. Meteorological data is downloaded from publicly available web servers. Finally satellite images have been acquired and processed under Google Earth Engine. All this information is loaded into the database management system.
2. *Initial data preparation.* An exhaustive preliminary analysis of all the data is carried out using graphical representation techniques, such as histograms. This allows to examine the data thoroughly and filter out those whose dispersion or variability may generate inconsistencies in the study.
3. *Anomaly identification.* Anomaly detection is performed using a classification algorithm that has the ability to determine, with a certain probability, whether a data record fits the expected distribution pattern. The objective at this stage is to identify outliers in our data.
4. *Data transformation.* In this section, the input data is modified before being inserted into the model. The purpose is to adapt the formats, units, rescaling or sorting by ranges, among other actions, to optimize their use in the predictive models. This ensures that the models can make efficient data use.
5. *Data aggregation.* The downloaded information is grouped by month; therefore, any other datasets that are added to the training data should be aggregated in the same way.
6. *Data integration.* In this analysis, a variety of data is available from different sources. In order to carry out the ML process, it is essential to unify all these data into a single source that serves as input for the predictive models. This requires the design of a suitable database that allows the input data to be properly stored in the system.

7. *Identification of how influential each input is on the target.* Before the model is generated, the level of influence of input data on the target attribute (Kg of olive fruit and olive oil) are analyzed in order to include or exclude them from predictive modeling.
8. *Regression algorithms applied.* Different regression algorithms are tested to perform the prediction. The objective of regression analysis is to determine the parameter values of a function that best fit an observational dataset. In addition to different families of regression functions, we also check different ways of measuring error.

3.3.1. Workflow process

The strategies employed are a series of mathematical operations that construct a design from the available information. To achieve this, a computational process first examines the data provided, detecting particular configurations of behaviour or specific directions. Through multiple iterations, it uses the results of this evaluation to determine the ideal settings that will shape the design. These settings are then used on the full data set to identify actionable trends and provide detailed statistical analysis. This in-depth analysis of the data is carried out using an analysis module integrated into Oracle Data Mining software. Selecting the most appropriate algorithm for our work is a significant challenge. The algorithms used in each phase and the workflow are described in Figure 3.

Once the dataset is available, the following step is the detection of data anomalies. This is to identify unusual cases in apparently homogeneous data. Anomalies detection is essential to recognise fraudulent activities, unusual data and any other rare events that have the potential to cause major changes, often these are difficult to detect. A classification algorithm is used for this purpose because these anomalies can be considered as a particular case of classification. A classifier of a class develops a profile that generally describes a typical case in the training data. Deviation from that profile is identified as an anomaly. Specifically, in this phase, the algorithm used has been the Support Vector Machine (SVM) algorithm, [44, 45, 46]. SVM is based on the fundamental idea of minimising the hypersphere of the single class of examples in the training data, and consider all other examples outside the hypersphere as outliers or outside the distribution of the training data. This algorithm generates a prediction and a probability for each case in the score data. If the prediction is 1, the case is considered typical. If the prediction is 0, the case is considered anomalous. This behavior reflects the

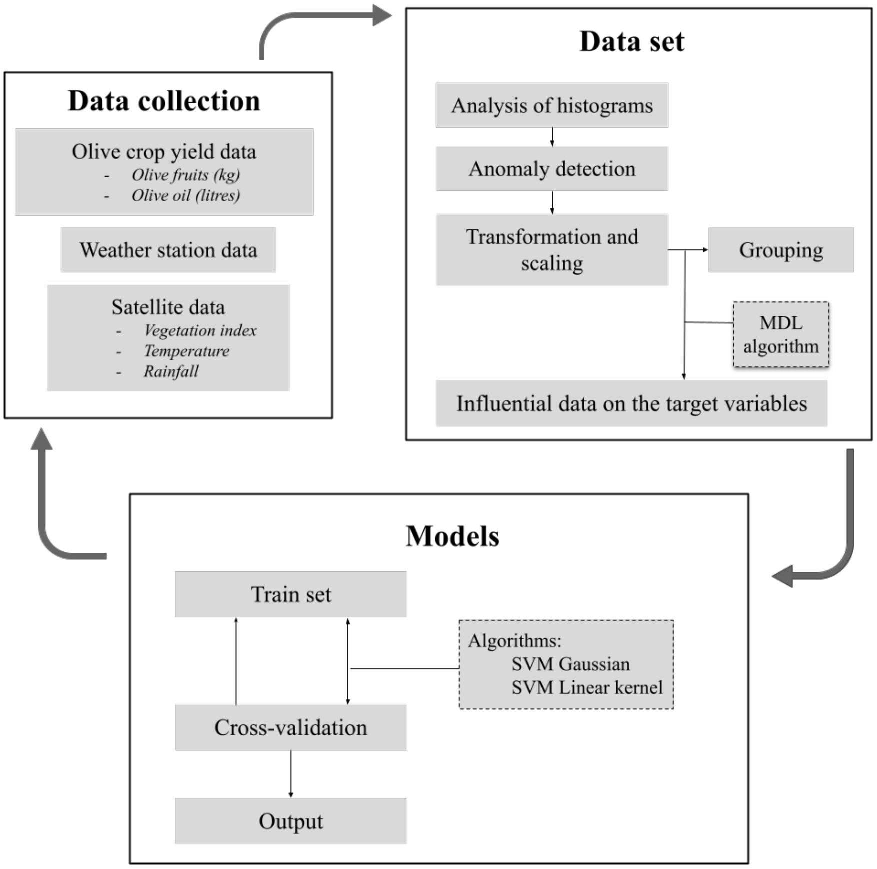

Figure 3: Workflow of the methodology used.

fact that the model is trained on data following a normal distribution. Also another linear regression algorithm as Generalised Linear Models (GLM) [44, 47] is used, but it has been discarded because in early tests it performed worse than SVM.

The next phase consists of determining the level of influence of the variables used on the target attribute. In this case, the Minimum Description Length (MDL) algorithm [48] is used. This algorithm holds that, given a limited set of observed data, the best explanation is the one that allows the greatest compression of the data. MDL considers each attribute as a simple predictive model of the target class. Model selection refers to the process

of comparing and ranking the simple predictive models. Running this algorithm produces a result in the range of −1 to 1. A value of 1 indicates that the features are highly related to the target, a value of 0 means that they are unrelated, and a negative value indicates that the feature is unrelated to the target, which could introduce noise into the study. In this analysis, only features with a weight greater than 0 are considered, discarding those with values equal to or less than 0.

Finally, regression analysis algorithms are used in the creation of the model, which focus on predicting the kilograms amount of olive crop and olive oil, both targets of this study. The choice to use regression is based on the fact that it is a data mining technique designed to anticipate numerical values over a continuous range. The regression process starts with a dataset in which the target values are already known. A regression algorithm calculates the desired value based on the predictors for each instance in that dataset. The relationships between the predictors and the target are encapsulated in a model that can then be applied to another dataset, where the target values are not known. In order to evaluate the effectiveness of regression models, calculations of various statistics are performed to assess the discrepancy between predictions and actual values. In a typical regression project, the historical dataset is divided into two parts: one to build the model and another to test the model. It is important to note that the year used for model testing is not part of the training dataset. In this study, SVM with Gaussian kernel and Linear kernel, respectively, have been used. SVM regression supports two types of kernels: the Gaussian kernel, which is used for non-linear regressions, and the linear kernel, which is suitable for linear regressions. SVMs work effectively on datasets containing many features, even if the number of cases to train the model is very small. There is no upper limit on the number of features that can be used; the restrictions only depend on the limitations imposed by the hardware used.

3.3.2. Predictor variables selected

Once the MDL algorithm is applied, the variables influencing both targets, quantity of Kg of olive crop and Kg of olive oil, are selected. These variables are shown in Table 7 calculated for each season and each year. As shown in the table, there are predictors whose values at various times in the previous, and also in the current year, are influential. This is the case, for example, for rainfall. In the crop prediction for the month of February (Winter), the values of rainfall both in the previous and current winter of the

<table>
<tr><td rowspan="3">Rainfall</td><td>Spring</td><td colspan="2" rowspan="2">Previous year</td></tr>
<tr><td>Summer - Autumn</td></tr>
<tr><td>Winter</td><td>Previous year</td><td>Actual year</td></tr>
<tr><td>Temperature</td><td>Winter</td><td colspan="2">Actual year</td></tr>
<tr><td rowspan="6">Vegetation index</td><td>ARVI</td><td colspan="2" rowspan="6">Actual year</td></tr>
<tr><td>NDMI</td></tr>
<tr><td>GCI</td></tr>
<tr><td>NDVI</td></tr>
<tr><td>SAVI</td></tr>
<tr><td>SIPI</td></tr>
</table>

Table 7: Predictor variables considered for each municipality, and by year and season indicated.

year of the prediction are considered to have an influence. The latter is often decisive for the crop yield, therefore its weight in the model is considered in this way.

The predictor variables must be categorized before applying the regression algorithms. For this purpose, the values are first normalized and then categorized by analyzing the histograms. The number of classes to be considered is set according to the variability of the data and then, a labeling process is applied.

The best predictors are those that best correlate the model. In this sense, numerous tests are carried out using different combinations of variables looking for the best set. By each predictors combination a model is obtained together with its corresponding Mean Absolute Error (MAE). This is calculated from each year's prediction, i.e. the difference between the predicted value and the real one. Afterwards, the total average of absolute errors of all the years is calculated, thus obtaining the consistency of the prediction for each set of predictors variables selected.

The MAE is the average of all absolute errors, and it is calculated as shown in Equation 1, where n is the number of errors, $\sum$ is the summation symbol which means ´´add them all up´´, and |xi - x| is the absolute errors.

$$MAE = \frac{1}{n}\sum_{\xi=1}^{n} |x_{\xi} - x| \tag{1}$$

3.3.3. Accuracy evaluation

After selecting the variables and ensuring all the information are accessible and standardized, the model is created. To gauge the model's effectiveness in predicting, we use the k-fold cross-validation technique [49]. This approach entails evaluating the model's predictive capability by isolating the data from a particular year and segregating it from the data used for model training. In the context of this research, it is utilized to validate the models' accuracy in predicting crop and oil production for a specific year. It's crucial to emphasize that the year in question has been deliberately omitted from the construction of both models, as mentioned above. This study encompassed the period from 2013 to 2021. Specifically, we have constructed a predictive model using data spanning seven years, followed by an evaluation of its reliability using data from the eighth year. In the cross-validation process, the prediction for each year is checked. Therefore, several models are generated, one for each year to be checked. The year to be predicted is always excluded from the training data and compared with the actual data.

As explained above, SVM models with two different kernels have been used: the Gaussian and the Linear kernels. After evaluation of these algorithms, the one that best fits the inherent characteristics of the data is selected. Subsequently, the final model is built using this chosen algorithm, with all data from 2013 to 2021 serving as the training set. In this initial results presentation phase, the generated models are subjected to cross-validation. Specifically, a model is developed for each year to be evaluated. For example, when evaluating the year 2021, the training data covers the years 2013 to 2020, deliberately excluding the 2021 data used to evaluate the effectiveness of the model. The same methodology is applied to the other models, where each year evaluated assumes the role of the test set, while being excluded from the training data. For example, in the case of testing the year 2017, the model training data comprises $[2013-2016]$ and $[2018-2021]$, with the exclusion of 2017.

4. Results and Discussion

4.1. Prediction using meteorological variables

The results obtained are highly dependent on the quality of the dataset. In this case, as the data come from different sources, it has been necessary to carry out an exhaustive review and a cleaning of outliers. This is essential to avoid introducing noise in the prediction and to be able to discern the most

<table>
<tr><th colspan="4" rowspan="3">Predictor variables</th><th colspan="4">Mean Absolute Error (%)</th></tr>
<tr><th colspan="2">Olive crop prediction</th><th colspan="2">Olive Oil prediction</th></tr>
<tr><th>SVM (Gaussian kernel)</th><th>SVM (Linear kernel)</th><th>SVM (Gaussian kernel)</th><th>SVM (Linear kernel)</th></tr>
<tr><td rowspan="3">Rainfall</td><td>Spring</td><td colspan="2" rowspan="2">Previous year</td><td rowspan="3">46 %</td><td rowspan="3">42 %</td><td rowspan="3">43 %</td><td rowspan="3">37 %</td></tr>
<tr><td>Summer - Autumn</td></tr>
<tr><td>Winter</td><td>Previous year</td><td>Actual year</td></tr>
<tr><td rowspan="3">Rainfall</td><td>Spring</td><td colspan="2" rowspan="2">Previous year</td><td rowspan="4">47 %</td><td rowspan="4">45 %</td><td rowspan="4">46 %</td><td rowspan="4">40 %</td></tr>
<tr><td>Summer - Autumn</td></tr>
<tr><td>Winter</td><td>Previous year</td><td>Actual year</td></tr>
<tr><td>Temperature</td><td>Winter</td><td colspan="2">Actual year</td></tr>
</table>

Table 8: Mean Absolute Error obtained in the model for the 97 municipalities as a whole, using only meteorological predictors. Results using the SVM algorithm with a Gaussian kernel and with a linear kernel.

influential set of predictors from those that have less influence. It should also be taken into account that the same dataset can be used to predict different targets, with the weight of each predictor in the model differing according to the target considered.

A first test is carried out considering only meteorological predictors, those are, rainfall and temperatures. The result of the cross-validation, Table 8, shows a mean absolute error in the prediction of olive crop quite similar to that obtained for olive oil.

In order to qualify the prediction obtained as good, the absolute error per municipality and per year is calculated. The total error of each test is obtained by adding up all the errors per year. In these first tests, high errors have been obtained. In general terms, it can be stated that the accuracy is low; however, it can be seen that using only rainfall gives slightly better results than if it is combined with the temperature values. It seems that the temperature has little influence on the model according to the results observed in Table 8. It can be seen that the temperature predictor not only does it not reduce the error of the model but slightly increases its value by introducing some noise. This may be due to the fact that temperature in the current winter is of little relevance to the yield of that year's crop.

Note that this result shows the average total error for the 97 municipalities considered. This error is also analyzed individually for each municipality. To do so, the MAE histogram is analyzed and it confirmed that a large number of municipalities have MAE values that are atypical with respect to the rest. These are a total of 19 municipalities. In this sense, and to guarantee the quality of the selection of predictors and the scope of our

Predictor variables				Mean Absolute Error (%)			
				Olive crop prediction		Olive Oil prediction	
				SVM (Gaussian kernel)	SVM (Linear kernel)	SVM (Gaussian kernel)	SVM (Linear kernel)
Rainfall	Spring	Previous year		27.71 %	27.14 %	27.11 %	27.44 %
	Summer - Autumn						
	Winter	Previous year	Actual year				
Rainfall	Spring	Previous year		27.93 %	27.20 %	27.15 %	27.45 %
	Summer - Autumn						
	Winter	Previous year	Actual year				
Temperature	Winter	Actual year					

Table 9: Mean Absolute Error obtained in the model without considering 19 municipalities with faulty data, using only meteorological predictors. Results using the SVM algorithm with a Gaussian kernel and with a linear kernel

prediction, another test is carried out by discarding these municipalities from the dataset. The result is shown in Table 9. Comparing Tables 9 and 10, it confirms the importance of proper data analysis and cleaning, as these 19 municipalities introduced significant noise into the model. Once these municipalities are removed from the dataset, the accuracy of the prediction improves considerably, around 30%.

Nevertheless, it is also observed that the SVM algorithm with Linear kernel, results in a better fitted model than using the Gaussian kernel. Overall, the low variability in the accuracy of the results using two different subsets of predictor variables may be due to the fact that these values as a whole differ little from one area to another. It may be necessary to use variables that better represent the state of olive orchards in each season and in each area. It is therefore considered appropriate to introduce vegetation indices among the combinations of predictor variables.

4.2. Prediction using vegetation index

The next test consists of introducing different vegetation indices into the set of predictors. In this case, after the thorough literature review described in previous sections, we have decided to consider those indices related to plant health and humidity values. In this sense, vegetation indices that provide values related to humidity can contribute with a better approximation of the state of the area after the weathering.

According to the literature review, a pre-selection of vegetation indices has been made. To determine the influence of each one on the model, the accuracy of each model is calculated by inserting rainfall values as predictors

**Predictor variables**					Mean Absolute Error (%)			
					Olive crop prediction		Olive Oil prediction	
					SVM (Gaussian kernel)	SVM (Linear kernel)	SVM (Gaussian kernel)	SVM (Linear kernel)
Previous year		Spring	Rainfall	ARVI	28.20%	26.80%	27.30%	26.67%
Previous year		Spring	Rainfall	NDMI	28.17%	26.80%	27.21%	26.78%
Previous year		Summer-Autumn	Rainfall	GCI	28.37%	27.17%	27.44%	27.26%
Previous year		Summer-Autumn	Rainfall	NDVI	28.13%	26.75%	27.27%	26.62%
Previous year	Actual year	Winter	Rainfall	SAVI	28.18%	26.63%	27.44%	26.55%
Previous year	Actual year	Winter	Rainfall	SIPI	28.31%	26.83%	27.33%	26.80%

Table 10: Prediction accuracy using meteorological predictors and each vegetation index individually.

**Predictor variables**					Mean Absolute Error (%)			
					Olive crop prediction		Olive Oil prediction	
					SVM (Gaussian kernel)	SVM (Linear kernel)	SVM (Gaussian kernel)	SVM (Linear kernel)
Previous year		Spring	Rainfall	ARVI + NDMI	28.64%	26.70%	27.55%	26.49%
Previous year		Spring	Rainfall	ARVI + SAVI	28.63%	26.59%	27.65%	26.30%
Previous year		Summer-Autumn	Rainfall	ARVI + NDMI + NDVI	29.14%	27.10%	27.98%	26.68%
Previous year		Summer-Autumn	Rainfall	ARVI + NDMI + GCI	29.02%	26.78%	27.82%	26.31%
Previous year	Actual year	Winter	Rainfall	ARVI + NDMI + SAVI	29.00%	26.65%	27.89%	26.21%
Previous year	Actual year	Winter	Rainfall	ARVI + NDMI + SIPI	29.07%	26.82%	27.88%	26.52%

Table 11: Prediction accuracy using meteorological predictors and subsets of vegetation index combinations.

next to each index separately. The results are shown in Table 10. The accuracy of the predictive model improves considerably by applying the Linear kernel, as the MAE is reduced to values of 26%. This accuracy in crop yield prediction, both for olives and olive oil is very useful for decision making. It should be borne in mind that this is an early prediction, before the start of the annual cycle of the olive tree and prior to the planning of investments to be made in tillage and phytosanitary products.

Once demonstrated the improvement of the predictive model by selecting the vegetation indices in the set of predictors, another test has been carried out using combinations of indices. Different combinations aree made using ARVI (Atmospheric Resistant Vegetation Index) as a fixed index. The reason is that it minimizes the atmospheric dispersion effects caused by aerosols such as rain, fog, dust, smoke or air pollution. Therefore, it can be considered as a correction to the NDVI index. Thus the selected subsets are shown in Table 11. In general terms, the improvement in prediction by integrating combinations of vegetation indices is not very significant, considering the effect on the region as a whole. A more detailed study, municipality by municipality, will probably allow to discern the most appropriate combination for each case. Nevertheless, this study confirms the influence of these indices and the potential predictive improvement of combining them.

5. Conclusions

This study presents a workflow methodology describing the steps followed in the analysis of the predictive calculation of olive crop yield at an early stage. The correct selection of the predictor variables and the quality of these variables are fundamental. For this, the knowledge about the type of crop and an exhaustive analysis of the influence of the variables considered guarantee to obtain an accurate predictive model. As has been shown, the availability of temporal data is essential. Only in this way a correct training of the regression model can be carried out. In this work it has been used the SVM algorithm with Linear and Gaussian kernel, the first being the one that provides the best predictive accuracy.

The integration of vegetation indices into the model improves crop yield prediction. This is due to the fact that a better diagnosis of the state of the plantation contributes to a good early prediction of its production. In this sense, satellite images have been fundamental in order to have sufficient temporality covering all the municipalities in the province of Jaén. The methodology developed is applicable to future works at different spatial scales, even at local or farm detail level. It is enough to have the historical harvest and meteorological data area under study, as well as UAV flights with multispectral sensors. The only limitation in this respect is the need to allow time to accumulate flights over the course of each year for as many years as possible.

[50]

Acknowledgments

This research has been partially funded through the research projects PREDIC_I-GOPO-JA-20-0006, cofinanced with the European Union FEDER, Instituto de Estudios Gienneses, and the Junta de Andalucía funds, also the projects PID2021-126339OB-I00 and TED2021-132120B-I00, funded by the Spanish Ministry of Science and Innovation. We are also grateful for the support provided by the Ministry for Ecological Transition and the Demographic Challenge, Spanish Government (AEMET, Agencia Estatal de Meteorología).

References

[1] Subdirección General de Análisis, Coordinación y Estadística, Anuario de estadística 2021, Tech. rep., Ministerio de Agricultura, Pesca y Alimentación (2021).

[2] S. Archana, P. S. Kumar, A Survey on Deep Learning Based Crop Yield Prediction, Nature environment and pollution technology 22 (2) (2023) 579–592, publisher: Technoscience Publications.

[3] P. Saini, B. Nagpal, Empirical Survey Analysis For Crop Yield Prediction & Identification Of Factors Affecting Yield Gaps, Journal of Pharmaceutical Negative Results 13 (2022) 1318–1329. doi:10.47750/pnr.2022.13.S05.207.

[4] S. Iniyan, V. A. Varma, C. T. Naidu, Crop yield prediction using machine learning techniques, Advances in Engineering Software 175 (2023) 103326. doi:https://doi.org/10.1016/j.advengsoft.2022.103326.

[5] R. Beulah, A Survey on Different Data Mining Techniques for Crop Yield Prediction, International Journal of Computer Sciences and Engineering 7 (1) (2019) 738–744, number: 1. doi:https://doi.org/10.26438/ijcse/v7i1.738744.

[6] G. Caruso, G. Palai, R. Gucci, S. Priori, Remote and Proximal Sensing Techniques for Site-Specific Irrigation Management in the Olive Orchard, Applied Sciences (Switzerland) 12 (3), type: Article (2022). doi:10.3390/app12031309.

[7] J. Brinkhoff, A. Schultz, L. A. Suarez, A. J. Robson, Olive Tree Water Stress Detection Using Daily Multispectral Imagery, in: 2021 IEEE International Geoscience and Remote Sensing Symposium IGARSS, 2021, pp. 5826–5829, iSSN: 2153-7003. doi:10.1109/IGARSS47720.2021.9553729.

[8] F. Solano, S. D. Fazio, G. Modica, A methodology based on GEOBIA and WorldView-3 imagery to derive vegetation indices at tree crown detail in olive orchards, International Journal of Applied Earth Observation and Geoinformation 83 (2019) 101912. doi:https://doi.org/10.1016/j.jag.2019.101912.

[9] D. Stateras, D. Kalivas, Assessment of olive tree canopy characteristics and yield forecast model using high resolution uav imagery, Agriculture (Switzerland) 10 (9) (2020) 1 – 13, type: Article. doi:10.3390/agriculture10090385.

[10] J. Oteros, F. Orlandi, H. García-Mozo, F. Aguilera, A. Dhiab, T. Bonofiglio, M. Abichou, L. Ruiz-Valenzuela, M. Del Trigo, C. Díaz De La Guardia, E. Domínguez-Vilches, M. Msallem, M. Fornaciari, C. Galán, Better prediction of Mediterranean olive production using pollen-based models, Agronomy for Sustainable Development 34 (3) (2014) 685–694. doi:10.1007/s13593-013-0198-x.

[11] F. Aguilera, L. Ruiz-Valenzuela, Forecasting olive crop yields based on long-term aerobiological data series and bioclimatic conditions for the southern Iberian Peninsula, Spanish Journal of Agricultural Research 12 (1) (2014) 215–224, section: Plant production (Field and horticultural crops). doi:10.5424/sjar/2014121-4532.

[12] A. Ben Dhiab, M. Ben Mimoun, J. Oteros, H. Garcia-Mozo, E. Domínguez-Vilches, C. Galán, M. Abichou, M. Msallem, Modeling olive-crop forecasting in Tunisia, Theoretical and Applied Climatology 128 (3) (2017) 541–549, number: 3. doi:10.1007/s00704-015-1726-1.

[13] H. F. Rapoport, S. B. M. Hammami, P. Martins, O. Pérez-Priego, F. Orgaz, Influence of water deficits at different times during olive tree inflorescence and flower development, Environmental and Experimental Botany 77 (2012) 227–233. doi:10.1016/j.envexpbot.2011.11.021.

[14] F. Aguilera, L. Ruiz-Valenzuela, A new aerobiological indicator to optimize the prediction of the olive crop yield in intensive farming areas of southern Spain, Agricultural and Forest Meteorology 271 (2019) 207–213. doi:10.1016/j.agrformet.2019.03.004.

[15] A. Lopez-Bernal, A. A. Fernandes-Silva, V. A. Vega, J. C. Hidalgo, L. Leon, L. Testi, F. J. Villalobos, A fruit growth approach to estimate oil content in olives, European Journal of Agronomy 123 (2021) 126206. doi:10.1016/j.eja.2020.126206.

[16] J. J. Cubillas, M. I. Ramos, J. M. Jurado, F. R. Feito, A Machine Learning Model for Early Prediction of Crop Yield, Nested in a Web Application in the Cloud: A Case Study in an Olive Grove in Southern Spain, Agriculture 12 (9) (2022) 1345, publisher: MDPI AG. doi:10.3390/agriculture12091345.

[17] L. Ortenzi, S. Violino, F. Pallottino, S. Figorilli, S. Vasta, F. Tocci, F. Antonucci, G. Imperi, C. Costa, Early Estimation of Olive Production from Light Drone Orthophoto, through Canopy Radius, DRONES 5 (4) (Dec. 2021). doi:10.3390/drones5040118.

[18] R. Pu, Hyperspectral Remote Sensing: Fundamentals and Practices, Remote Sensing Applications Series, CRC Press, 2017.

[19] P. Catania, E. Roma, S. Orlando, M. Vallone, Evaluation of Multispectral Data Acquired from UAV Platform in Olive Orchard, Horticulturae 9 (2), type: Article (2023). doi:10.3390/horticulturae9020133.

[20] M. Atik, B. Akdemir, Spatial Variability of NDVI and SPAD for Variable- and Fixed-Rate Fertilization in Olive Orchards, Erwerbs-Obstbau 64 (4) (2022) 647–655. doi:10.1007/s10341-022-00748-w.

[21] E. Roma, V. A. Laudicina, M. Vallone, P. Catania, Application of Precision Agriculture for the Sustainable Management of Fertilization in Olive Groves, Agronomy 13 (2), type: Article (2023). doi:10.3390/agronomy13020324.

[22] B. Boshkovski, G. Doupis, A. Zapolska, C. Kalaitzidis, G. Koubouris, Hyperspectral Imagery Detects Water Deficit and Salinity Effects on Photosynthesis and Antioxidant Enzyme Activity of Three Greek Olive Varieties, Sustainability (Switzerland) 14 (3) (2022). doi:10.3390/su14031432.

[23] F. López-Granados, M. Teresa Ǵomez Casero, J. M. Peña-Barragán, M. Jurado Exp\'osito, L. García Torres, Classifying irrigated crops as affected by phenological stage using discriminant analysis and neural networks, Journal of the American Society for Horticultural Science 135 (5) (2010) 465 – 473, type: Article. doi:10.21273/jashs.135.5.465.

[24] T. Yildirim, Y. Zhou, K. C. Flynn, P. H. Gowda, S. Ma, D. N. Moriasi, Evaluating the sensitivity of vegetation and water indices to monitor drought for three Mediterranean crops, Agronomy Journal 113 (1) (2021) 123–134, _eprint: https://acsess.onlinelibrary.wiley.com/doi/pdf/10.1002/agj2.20475. doi:https://doi.org/10.1002/agj2.20475.

[25] E. Moltó, Fusion of Different Image Sources for Improved Monitoring of Agricultural Plots, Sensors 22 (17), type: Article (2022). doi:10.3390/s22176642.

[26] F. Lima-Cueto, R. Blanco-Sepúlveda, M. Gómez-Moreno, F. Galacho-Jiménez, Using vegetation indices and a UAV imaging platform to quantify the density of vegetation ground cover in olive groves (Olea Europaea L.) in Southern Spain, Remote Sensing 11 (21) (2019). doi:10.3390/rs11212564.

[27] G. Modica, G. Messina, G. D. Luca, V. Fiozzo, S. Praticò, Monitoring the vegetation vigor in heterogeneous citrus and olive orchards. A multiscale object-based approach to extract trees' crowns from UAV multispectral imagery, Computers and Electronics in Agriculture 175 (2020) 105500. doi:https://doi.org/10.1016/j.compag.2020.105500.

[28] V. Papić, N. Bugarin, J. Gugić, On Olive Groves Analysis using UAVs, in: 2021 29th International Conference on Software, Telecommunications and Computer Networks, SoftCOM 2021, 2021, type: Conference paper.

[29] G. Rallo, M. Minacapilli, G. Ciraolo, G. Provenzano, Detecting crop water status in mature olive groves using vegetation spectral measurements, Biosystems Engineering 128 (2014) 52–68. doi:https://doi.org/10.1016/j.biosystemseng.2014.08.012.

[30] P. Battista, E. Bellini, M. Chiesi, S. Costafreda-Aumedes, L. Fibbi, L. Leolini, M. Moriondo, B. Rapi, R. Rossi, F. Sabatini, F. Maselli, Estimating the effect of water shortage on olive trees by the combination of meteorological and Sentinel-2 data, European Journal of Remote Sensing 56 (1), type: Article (2023). doi:10.1080/22797254.2023.2194553.

[31] T. Dindaroğlu, M. Kılıç, E. Günal, R. Gündoğan, A. E. Akay, M. Seleiman, Multispectral UAV and satellite images for digital soil modeling with gradient descent boosting and artificial neural network, Earth Science Informatics 15 (4) (2022) 2239 – 2263, type: Article. doi:10.1007/s12145-022-00876-7.

[32] S. Arenas-Castro, J. Gonçalves, SDM-CropProj – A model-assisted framework to forecast crop environmental suit-

ability and fruit production, MethodsX 8 (2021) 101394. doi:https://doi.org/10.1016/j.mex.2021.101394.

[33] X. Xu, P. Gao, X. Zhu, W. Guo, J. Ding, C. Li, M. Zhu, X. Wu, Design of an integrated climatic assessment indicator (ICAI) for wheat production: A case study in Jiangsu Province, China, Ecological Indicators 101 (2019) 943–953. doi:10.1016/j.ecolind.2019.01.059.

[34] O. Fabio, S. Carlo, B. Tommaso, R. Luigia, R. Bruno, F. Marco, Yield modelling in a Mediterranean species utilizing cause–effect relationships between temperature forcing and biological processes, Scientia Horticulturae 123 (3) (2010) 412–417, number: 3. doi:10.1016/j.scienta.2009.09.015.

[35] H. García-Mozo, R. Perez-Badía, C. Galán, Aerobiological and meteorological factors' influence on olive (Olea europaea L.) crop yield in Castilla-La Mancha (Central Spain), Aerobiologia 24 (1) (2008) 13–18, number: 1. doi:10.1007/s10453-007-9075-x.

[36] N. Oses, I. Azpiroz, S. Marchi, D. Guidotti, M. Quartulli, I. G. Olaizola, Analysis of copernicus' era5 climate reanalysis data as a replacement for weather station temperature measurements in machine learning models for olive phenology phase prediction, Sensors (Switzerland) 20 (21) (2020) 1 – 22, type: Article. doi:10.3390/s20216381.

[37] C. Chandana, G. Parthasarathy, A comprehensive survey of classification algorithms for formulating crop yield prediction using data mining techniques, in: Proceedings of 2020 IEEE International Conference on Technology, Engineering, Management for Societal Impact Using Marketing, Entrepreneurship and Talent, TEMSMET 2020, 2020, type: Conference paper. doi:10.1109/TEMSMET51618.2020.9557403.

[38] M. Atef, A. Khattab, E. A. Agamy, M. M. Khairy, Deep Learning Based Time-Series Forecasting Framework for Olive Precision Farming, in: Midwest Symposium on Circuits and Systems, Vol. 2021-August, 2021, pp. 1062 – 1065, type: Conference paper. doi:10.1109/MWSCAS47672.2021.9531929.

[39] H. Ribeiro, M. Cunha, I. Abreu, Quantitative forecasting of olive yield in Northern Portugal using a bioclimatic model, Aerobiologia 24 (3)

(2008) 141, number: 3. doi:10.1007/s10453-008-9094-2.
URL https://doi.org/10.1007/s10453-008-9094-2

[40] T. Poblete, J. Navas-Cortes, C. Camino, R. Calderon, A. Hornero, V. Gonzalez-Dugo, B. Landa, P. Zarco-Tejada, Discriminating Xylella fastidiosa from Verticillium dahliae infections in olive trees using thermal- and hyperspectral-based plant traits, ISPRS Journal of Photogrammetry and Remote Sensing 179 (2021) 133–144. doi:10.1016/j.isprsjprs.2021.07.014.

[41] F. Maselli, M. Chiesi, L. Brilli, M. Moriondo, Simulation of olive fruit yield in Tuscany through the integration of remote sensing and ground data, Ecological Modelling 244 (2012) 1–12. doi:https://doi.org/10.1016/j.ecolmodel.2012.06.028.

[42] F. Carreño-Conde, A. E. Sipols, C. Simón, D. Mostaza-Colado, A forecast model applied to monitor crops dynamics using vegetation indices (Ndvi), Applied Sciences (Switzerland) 11 (4) (2021) 1 – 25, type: Article. doi:10.3390/app11041859.

[43] J. D. Sánchez Martínez, A. Garrido Almonacid, Dinámica Temporal Y Caracterización Territorial Del Olivar En La Provincia De Jaén, 1st Edition, Caja Rural de Jaen, Barcelona y Madrid Sdad Coop Credito, España, 2021.

[44] A. J. Dobson, An introduction to generalized linear models, 2nd Edition, Chapman & Hall/CRC texts in statistical science series, Chapman & Hall/CRC, Boca Raton, 2002.

[45] R. Chalapathy, A. K. Menon, S. Chawla, Anomaly Detection using One-Class Neural Networks, arXiv:1802.06360 [cs, stat]ArXiv: 1802.06360 (Jan. 2019).

[46] P. Oza, V. M. Patel, One-Class Convolutional Neural Network, IEEE Signal Processing Letters 26 (2) (2019) 277–281, number: 2 Conference Name: IEEE Signal Processing Letters. doi:10.1109/LSP.2018.2889273.

[47] B. M. Bolker, M. E. Brooks, C. J. Clark, S. W. Geange, J. R. Poulsen, M. H. H. Stevens, J.-S. S. White, Generalized linear mixed models: a practical guide for ecology and evolution, Trends in Ecology & Evolution 24 (3) (2009) 127–135, number: 3. doi:10.1016/j.tree.2008.10.008.

URL https://www.sciencedirect.com/science/article/pii/S0169534709000196

[48] P. D. Grünwald, J. I. Myung, M. A. Pitt, Advances in Minimum Description Length: Theory and Applications, Neural Information Processing series, A Bradford Book, Cambridge, MA, USA, 2005, series Editors: _:n652.

[49] J. D. Rodriguez, A. Perez, J. A. Lozano, Sensitivity Analysis of k-Fold Cross Validation in Prediction Error Estimation, IEEE Transactions on Pattern Analysis and Machine Intelligence 32 (3) (2010) 569–575, number: 3 Conference Name: IEEE Transactions on Pattern Analysis and Machine Intelligence. doi:10.1109/TPAMI.2009.187.

[50] Distribución general de la superficie (Oct. 2020).
URL https://www.ine.es/jaxi/Tabla.htm?path=/t01/p044/a2016/ccaa00/l0/&file=0102.px&L=1
