# Supplementary material for: Improving early prediction of crop yield in Spanish olive groves using satellite imagery and machine learning
Source: PLoS One. 2025 Jan 15;20(1):e0311530. doi: 10.1371/journal.pone.0311530 (PMC11734994; doi:10.1371/journal.pone.0311530)
Supplement: Attachment — Submitted filename: Review report PONE-D-24-04068.pdf [file pone.0311530.s002.pdf]

Review report:

I have read the manuscript entitled " Early season crop yield prediction using temporal data and satellite imagery with machine learning. A new avenue for planning in Spanish olive groves." The manuscript can be considered for publication after the following questions and recommendations:

1- The abstract must clearly articulate the originality of the investigation.
2- The authors did not specify the method used to determine the sample size.
3- If the authors draw A flowchart to the workflow in the methodology, the part will help link the audiences to how this method advances current knowledge and will help the audiences follow the results quickly.
4- The researchers need to mention the experimental design they used in this manuscript to help the readers follow the results and the conclusions.
