# Supplementary material for: Improving early prediction of crop yield in Spanish olive groves using satellite imagery and machine learning
Source: PLoS One. 2025 Jan 15;20(1):e0311530. doi: 10.1371/journal.pone.0311530 (PMC11734994; doi:10.1371/journal.pone.0311530)
Supplement: Attachment — Submitted filename: Response to Reviewers.pdf [file pone.0311530.s003.pdf]

Reviewer 1#:

Reviewer comment: The topic seems interesting and it seems that the authors have done a good job. However, the paper is very lengthy and unnecessary details are added which makes the paper boring and distracting. The extra details may be good for inclusion in thesis but not in a research paper.

Author response: We appreciate your valuable comments on our manuscript, which have helped to improve the quality of our work.

The Introduction and State of the Art sections have been rewritten and summarized, especially the second one to focus more on the problem and the solution we propose.

Reviewer comment: Further, the model does not seem to achieve a very good performance. 26% error seems to be too high. I will recommend the authors to try some other models such as ANN, Random Forest, or even considering deep learning models is not a bad choice.

Author response: At first glance it may seem that the prediction error is high, but it should be borne in mind that the time of year at which such a prediction is made. These data are predicted just at the end of the previous year's harvest season, when there is still no physical evidence in the field to indicate what the next year's harvest will be like. That is, long before there is any pollen or flowering on the farm.

Recent works, such as (Aquino et al., 2023), predict olive fruit with a low predictive error, 2.64%, but this data is provided from computer analysis of visible field data, i.e. two months prior to fruit harvesting. Others as (Nasiri et al., 2024) performs fruit prediction from tree root analysis. In this case, it is necessary the installation of sensors in the farm and take several samples throughout the year. However, our work makes the prediction eight months, for the early harvest, from the beginning of the first campaigns of the year, with remote and non-invasive techniques.

This issue has been clarified in the text and this paragraph has been added to State of the art section.

Reviewer comment: Some other point that needs consideration are;
1. The title requires reconsideration. Why " A new avenue for planning in Spanish olive groves" is important.

Author response: This comment has been considered and the title has been changed. The new one is “Improving early prediction of crop yield in Spanish olive groves using satellite imagery and machine learning”. This new avenue is important for olive groves and particularly in Spain because there is no literature evidence that any research has been carried out on crop yield prediction at such an early stage of the olive crop year. Most of the

research is based on data from evidence already present in the field, such as pollen counts, aerobiological factors, etc. However, this work makes the prediction just when the harvesting of the previous year's crop has just finished. When no measurable evidence already exists in the field.

Reviewer comment: 2. the first few paragraphs of Section 2 is not about the state of the art but can be a good motivation for the work. Please reduce the length of the text and focus on the relevant works. I will also recommend removing some irrelevant works and add some recent works instead.

Author response: We have considered this suggestion, and some portions of this text have been moved to the Introduction section. In order to reduce the length of this section, some sentences have been removed or simplified, especially those not relevant to the work or already mentioned elsewhere.

Reviewer comment: 3. The overall language quality is reasonable, however, at some points, it requires proof read. For instance Line 161 "... on Feb ..." => "... in Feb...". There are other such instances as well.

Author response: These suggestions have been taken into account and we have solved these language issues.

Reviewer comment: 4. Line 331, "This information is extracted from .... " what information are you referring to?

Author response: We refer to the information just described in the previous paragraph. However, in order to improve the comprehensibility of the text, we have modified the drafting of the text.

Reviewer comment: Recommendation:
I like the topic and the amount and diversity of data that is being collected. However, the paper in current form and with the current models is not interesting. I will recommend a complete re-write, making it crisp and to the point. Most importantly, I will recommend implementation more ML models such as ANN, random forest and deep learning models.

Author response:

The authors welcome the reviewer's suggestions for improving the article. As recommended, we have inserted in the text the tests carried out with other regression models and it has been explained the advantages and disadvantages of each have been explained, including the use of deep learning. The text added and the analyses performed have been added previously to section 3.3.1.

“The choice of crop prediction algorithms depends mainly on the ability to handle large and complex data sets with multiple input variables, which is our case. It also depends on the need to model linear or non-linear relationships and whether the types of data to be predicted are continuous or categorical. In addition, algorithms are required that are able to capture the interactions between the various agricultural factors that affect the yield of the olive crop, such as yield data from previous years, weather conditions and the spectral response of the crop as a function of soil properties, plant condition and agricultural practices. Our variable to be predicted, the crop yield, is a numerical and continuous data, so regression algorithms were applied, and within these, Random Forest, GLM, SVM with linear kernel and Gaussian were selected, and finally, tests with Neural Networks were also carried out. The reasons are that Random Forest, GLM, SVM with linear and Gaussian kernels, and Neural Networks share the ability to model complex nonlinear relationships through specific structures and transformations. Unlike regression algorithms such as Gradient Boosting Machines, which sequentially correct prior errors, or K-Nearest Neighbors, which depend on data proximity, these models can address a greater diversity of patterns in the data. In addition, they differ from Deep Learning techniques, which use multiple layers to extract progressively more abstract features and are more suitable for large volumes of data and unstructured features such as images and text.”

Also, in the 3.3.1 section has been added the following paragraph:

“As described in the State of the Art section, the type of target in this study makes regression algorithms the ideal ones to perform the regression, however, it is initially unknown which one will work best. To select the best one, it is necessary to perform preliminary tests and analyze the errors of each one. In this case, the preliminary tests completely ruled out the use of the GLM, since the resulting errors were not tolerable. The reason for such results is that it is a pure linear algorithm, prevailing nonlinear relationships between the variables of the dataset. Random Forest, SVM (with linear and Gaussian kernel) and neural networks were then applied. Although in their basic forms some of these algorithms inherently handle linear separations (such as linear SVM), all three can be extended or inherently possess the ability to model nonlinear relationships”

Reviewer comment: For guidelines, I found the following papers as good examples to follow;

1. doi.org/10.1002/agj2.21382
2. doi.org/10.1016/j.eswa.2021.115511
3. doi.org/10.1016/j.rse.2018.11.032

Some of these works may not be regression based problems, but it give a good idea about writing such a paper.

Author response; We have considered these papers for being analyzed and being included into the bibliography as suggested:

#1: This paper is about classification of tobacco using multispectral time series data from two different remote sensing sources, Sentinel-2 and Planet Scope. We don’t have a specific section for classification. However, it is an example of very compact and summarized work. We we take note of its structure

#2: This paper has been cited. It uses regression analysis and it is interesting for us since they manage MODIS satellite data for temperature in a similar way to our method.

#3: This paper is about classification of different crops and it is focused on the way of extracting temporal features from the temporal series. It is not the aim of this paper, but I understand the paper structure in which different machine/deep learning methods are compared.

Reviewer 2#:

Reviewer comment: The state of the art and conclusions should discuss the environmental and socio-economic challenges of olive production as a socio-ecological system. Olive production data from Spain should be compared with other regions of the world as well as their problems, challenges and alternatives (a SWOT analysis may be useful).

Author response: We have rewritten most sections of the paper. The first three paragraphs of the paper now refer to the economic impact of the drop in production regarding other countries. Additionally, the first paragraph in Section 2 reflects the particular benefits of an early crop prediction for all those agents involved in the olive sector.

Reviewer comment: The authors do not discuss the implications of their research for sustainable development.

Author response: This is an interesting recommendation because although we have focused our aim into the economic impact, the environmental issue is also important and we think it's really related with our work. We have included a new paragraph in Section 2 (3º paragraph). As we analyze different vegetation indices, we are able to know the status of the olive crop health and act consequently. This can help to adjust the use of pesticides or fertilizers.

Reviewer comment: The MS is very extensive. The authors could reduce some sections such as the state of the art, spectral indices among others and strengthen the discussion and conclusions.

Author response: We have rewritten most parts of the paper and reduced the text as much as possible. However, we have concluded that there are numerous key points to indicate in order to fully understand the framework

Reviewer 3#:

Reviewer comment: Overall, the authors have conducted an excellent study to examine prediction analysis for olive trees with multiple dataset. However, the following information is not clear in the publication. Therefore, the paper needs improvement and should be written in a more understandable manner.

Author response: Many sections and paragraphs have been rewritten according to the reviewer's comments, mostly trying to clarify objectives and methodology.

Reviewer comment: 1. The introduction section should be expanded within the scope of methodology and prediction analyses.

Author response: The introduction section is now focused on the objective of the paper, the methodology followed and the key findings of the paper.

Reviewer comment: 2. In the final paragraph of the introduction, a brief overview should be provided on how the low spatial resolution of satellite images used in the classification of a single tree or tree clusters was addressed in the study.

Author response: We have added a new paragraph at the end of Introduction section as a brief description of methodology and we claim that resolution is made at municipal level, but only considering those plots cultivated with olive trees, as the municipal government provides this information through SIGPAC (https://www.juntadeandalucia.es/agriculturaypesca/sigpac/index.xhtml). Later, in Section 3 this information is explained in detail.

Reviewer comment: 3. The Table 4 should be within the content of section 3.2.3.

Author response: The authors appreciate the reviewer's suggestion but consider it more convenient that Table 4 is cited in section 3.3.2 which describes the variables used as predictors and their justification.

Reviewer comment: 4. The visual representation of processed and utilized satellite image data with the Google Earth Engine system should be provided.

Author response: We have introduced a new image in the text (Figure 4 in the new version). This image shows a map of the province of Jaén and the calculation of the NDVI index on areas with olive groves. Some portions of the code are also shown.

Reviewer comment: 5. In “Data Transformation” section, It should be written which data formats, scales, etc. have been modified

Author response: This suggestion has been considered and the following paragraph has been added to the “Data Transformation” section:

“As an example, the specific transformations that have been made to the data are those that are typical of multi-source data integration. In the case of the meteorological data, file format conversions have been necessary, and in the case of the vegetation indices, a classification and subsequent labelling of the ranges has been required in order to be able to insert them into the models.”

Reviewer comment: 6. Tables 10 and 11 should be explained in more detail in “4.2. Prediction using vegetation index”.

Author response: This suggestion has been considered and, in order to clarify the analysis, these tables have been replaced by the figures 7 and 8 (figures 6 and 7 in the last version of the manuscript)

Reviewer comment: 7. Why is prediction not made solely with vegetation indices apart from meteorological data? If feasible, it would be beneficial to make a prediction solely based on vegetation indices and share the outcome.

Author response: This test was already carried out, but the results worsened considerably. The vegetation indices improve the prediction but taken in isolation they worsen the model fit. Meteorological variables and especially rainfall is a very influential predictor.

Reviewer comment: 8. It is understood from the publication that prediction error values have been determined. However, upon reviewing the introduction and publication title, it is implied that the quantity of the crop to be harvested for the studied area will be determined. To address this complexity, explanations need to be provided within the text.

Author response: The authors have been considered this suggestions and this issue has been clarified in the Introduction section adding the next paragraph:

“The olive crop yield is understood as the two variables described above, the number of kilograms of olives to be harvested and the amount of olive oil obtained. It is necessary to understand that both variables are related but not in direct proportionality.”

Reviewer 4#:

This work demonstrates the potential for advanced technologies and methodologies to significantly benefit agricultural management and decision-making processes. The methodological rigour explored a d the resulting prediction accuracy is worth noting.

Reviewer 5#:

Reviewer comment: 1- The paper title is too long, so it need to be revised.

Author response:

This suggestion has been taken into account and the title has been revised.

The new title is"Improving early prediction of crop yield in Spanish olive groves using satellite imagery and machine learning"

Reviewer comment: 2- The abstract is not highlighted the paper findings well and need to be rewritten.

Author response:

This suggestion has been considered and the abstract has been rewritten. The changes made to the text are as follows (added in red)

In the production sector, the usefulness of predictive systems as a tool for management and decision-making is well known. In the agricultural sector, a correct economic balance of the farm depends on making the right decisions. To this end, having information on crop yields for the next harvest when the current crop has just been harvested is an extraordinary help. At that time, there is no visible indication in the field of how productive or unproductive the next harvest will be. Numerous predictive models infer accurate crop yield data from aerobiological variables and pollen analysis; this is around spring, in the middle stage of the crop year, when planning and investments are already done. The aim of this study is to anticipate accurate crop yield data at an early stage of the cropping season. In the case of olive groves in Spain, this period is in February. This work is developed for an entire province, Jaen, belonging to the region of Andalusia, in Southern Spain. The methodology uses Machine Learning algorithms together with an exhaustive analysis of predictor variables. Temporal data come from public web services, such as spatial data infrastructures of some state agencies. The processing of the satellite imagery is carried out by the geospatial processing service Google Earth Engine. The result is the early prediction of kilograms of both olive fruit ~~crop~~ and olive oil crop, eight months prior to the beginning of the first harvesting campaigns of the year, with an average absolute error of prediction better than 26%. The relevance of this work is the early availability of predicted crop yield data together with the multi-scale applicability of the predictive models. This makes this model a useful tool for all the agents involved in olive grove management. From farmers, to agricultural technicians, researchers and scientists dedicated to the study of the olive tree, to governmental institutions and agricultural associations that provide technical support,

advice and regulation to ensure responsible practices and the long-term viability of the olive industry.

Reviewer comment: 3- Authors did not mention the official sources and agencies from where they obtained the dataset.

Author response:

All data used in this study is publically available and it is obtained from different sources. All this information is indicated in their respective sections:

- Vegetation indices from satellites (Section 3.2.2)
- Weather information from satellites (Section 3.2.3)
- Plots and land use from a public administration of Andalusia (Section 3.1)

No additional sources of information are necessary.

Reviewer comment: 4- Absolute error is a measure of how far off' a measurement is from a true value or an indication of the uncertainty in a measurement. So 26% means authors are very far off from the true value.

Author response:

It is true that a predictive error of 26% is high in a prediction. However, the error must be analyzed in the context of the research in question. Firstly, this is a prediction at a stage prior to the start of the crop's vegetative period, in Spain this period begins in spring and the prediction is made two months before it starts. On the other hand, the scale of the study has to be considered. In another study (Cubillas et al., 2022) , a prediction was made with fewer predictor variables and for a working area relating to a single olive orchard farm. In this case, 97 municipalities in the whole province of Jaén were covered, which is an area of 13496 km2.

Reviewer comment: 5- The paper novelty is low, all models and tools used are exist in the state-of-the-art.

Author response: The major contribution of this paper is the specific moment in which prediction is done (Early prediction before flowering). None of the revised bibliography provides results at this time, even 10 months before next harvest. The second contribution is the set of input variables used, combining meteorological and vegetation indices as well as the historic crop yield data. We have introduced a new image (Figure 1) in which

On the other hand, the authors agree with the reviewer that the models and techniques used already exist in the state of the art. However, the novelty of this paper is the methodology implemented and the aim of the research. This involves the capture, preparation and integration of multi-source and multi-scale data. Furthermore, the data is from public governmental agencies, which ensures the continuity and

extension of the training data set. In order to make a prediction of crop yield data at a very early stage of the olive crop year, research that has not been addressed in the literature.

Reviewer comment: 6- To reflect the robustness of author proposed method, a comparison with two or more state of art models is preferred.

Author response:

This suggestion has been taken into consideration and three more regression models have been proposed (Random Forest, Generalized Linear Regression (GML) and Neuronal Network (NN) and compared with the ones considered previously. In addition, their use has been justified and why they have been discarded in the final model. All this discussion have been included in the text from Line 688 to Line 709

None of the literature revised uses the same dataset mentioned above, and neither of them performs early prediction. We have highlights this fact by introducing Figure 1 and along the text of the paper.

Reviewer comment: 7- I doubt it can have said to be a journal paper with significant contributions.

Author response: It's possible that the contribution of this paper was not sufficiently clear in the previous version of the paper. We have highlighted this main contribution along the text and specially in the Introduction Section.

Reviewer #6

Reviewer comment: Paper is welln written but methodology section can be improved by providing figure of methodology working to improve the understanding.

Author response:

This suggestion has been considered and additional figures have been added to the text and others have been modified. Specifically:

- In the "State of the art" section Figure 1 has been added in order to emphasise the novelty of the research.
- Figure 4 has been modified in order to clarify the understanding of the workflow of the methodology used.
- Figure 5 has been added into the Results and Discussion section in order to clarify the comparison between models using different combinations of predictor variables.

Reviewer comment: Improve the English and reduce jorgans to.improve reader understanding.
Improve the conclusion section of the paper by more clearing the expected outcomes received from this research

Author response:

This suggestion has been considered and the text has been revised and jorgans have been removed and the Conclusions section has also been revised. The following paragraph has been added:

"In this research, predictive models of crop yield at an early stage of the olive growing season in Spain has been generated. This is before spring, just when the previous year's harvesting season has just ended and there is still no evidence in the field of how productive the next season will be. The targets of the predictive models are both olive fruit yield and oil yield. These are continuous numerical data, hence regression algorithms have been used when applying ML. It has been confirmed the SVM algorithm with Gaussian kernel provides the best predictive accuracy".

Reviewer 7#:

Review comments

This work mainly employed SVM to predict the olive crop and oil yield using satellite data. However, there are significant issues throughout this manuscript thus leading it is unsound at current version.
Significantly, this work fails to offer compelling evidence of the success of the main prediction task, which is using an SVM regression model for crop yield prediction. It only presents Mean Absolute Error (MAE), which is insufficient for evaluating the model's performance effectively (or cannot fully capture the severity of prediction errors) within the specific context of crop yield prediction. Additionally, several steps in machine learning lack clarity without further elaboration even the methods section hints at some clues. Specifically, normalization or standardization, anomaly detection, hyperparameter tuning, and feature selection require thorough calibration and detailed explanation.
Thus, I cannot currently endorse this manuscript for publication in this journal as it requires substantial improvements to meet the promised standards in academic research.
My detailed review comments are listed below.

Author response:

In order to measure error we have added to the Mean Absolute Error (MAE) the Root Mean Squared Error (RMSE). In order to clarify this issue in the text in section 3.3.2 has been added the following text:

“The RMSE, Equation 2, is also calculated for each model, because the two metrics are complementary. MAE measures average absolute errors, offering robustness against outliers and straightforward interpretability. However RMSE emphasizes larger errors due to its squaring step, making it sensitive to outliers and suitable for scenarios where large errors are critically undesirable. RMSE can disproportionately affect model evaluation due to its emphasis on larger errors”.

Also, in order to clarify the steps in ML the following text has been added the “Workflow process” section

“As described in the State of the Art section, the type of target in this study makes regression algorithms the ideal ones to perform the regression, however, it is initially unknown which one will work best. To select the best one, it is necessary to perform preliminary tests and analyze the errors of each one. In this case, the preliminary tests completely ruled out the use of the GLM, since the resulting errors were not tolerable. The reason for such results is that it is a pure linear algorithm, prevailing nonlinear relationships between the variables of the dataset. Random Forest, SVM (with linear and Gaussian kernel) and neural networks were then applied. Although in their basic forms some of these algorithms inherently handle linear separations (such as linear SVM), all three can be extended or inherently possess the ability to model nonlinear relationships”.

The study incorporates data preprocessing, anomaly detection, and transformation steps to optimize the input for predictive modeling. It also employs regression and analysis and evaluates the influence of various predictors on the target variables (kg of live fruit and olive oil). SVM is used in the pre-detection of anomalies in the data. In this case, a complexity criterion is set from which the algorithm does not consider in the calculations those data that exceed a threshold. From there, to generate the prediction, the SVM performs an unsupervised prediction of non-categorical numerical values.In this sense the default parameters were used by the system (Oracle Data Mining). They are: Standard Deviation 2.028.306, Complexity Factor 0.589921, Kernel Function Gausiano, Algorithm Name Support Vector Machine, Active Optimisation Enable, Automatic Preparation Enable, SVMS_EPSILON 0.040708, Core Cache Size 50,000.000 and Tolerance 0.001. These last details have been added in 4.1 section.

1. Introduction

Reviewer comment: 1.1 The title needs revision: there's no need to incorporate ‘temporal data’, as the result in this manuscript has not addressed the importance of including temporal trends through any method involving modelling in the time dimension (e.g., ARIMA, RNN, LSTM). In terms of the subtitle, it is preferable to phrase it as follows: ‘A case study of olive groves in Jaden Span’ as the insufficient discussion or analysis regarding the planning implications in this manuscript.

Author response: This suggestion has been taken into account and the title has been revised. The new title is”Improving early prediction of crop yield in Spanish olive groves using satellite imagery and machine learning”

Reviewer comment: 1.2 The research gap and the research question should be clearly addressed in introduction section.

Author response: The Introduction section have been almost completely rewritten and we think that now it reflects better the main contribution and the methodology used

Reviewer comment: 1.3 The early crop prediction requires a clearer and more detailed definition following a background overview of the olive growing season provided in this section. The early crop prediction refers to forecasting tasks conducted in the early stages of the season, typically around February (in the case of olive crop), utilizing data from previous years? Also, a discrimination of early prediction and late prediction can be included in this section.

Author response: We have formally defined the concept of "Early Prediction" in Introduction section (paragraph 4). This makes sense for the northern hemisferic.

Reviewer comment: 1.4 In line 67, a typo: 'this q work'

Author response: Thank you very much. Solved.

2. Related work (State of art)

Reviewer comment: 2.1 This section requires further revision as it encompasses multiple aspects. First, the description of the Spanish olive case, including the importance of early prediction of olive yield, could be incorporated into the case study section (or study area). Second, the review of key factors in crop yield prediction needs to be organized to list variables and their related indices more succinctly. Integrating this information would enhance the informativeness of Table 2.

Author response: We have rewritten most of the text in this section and the following paragraph has been added to the Introduction section:

"In olive growing, is considered to be that which precedes pollination, that is, in the months of February and March (in the northern hemisphere).In the particular case of the olive grove, most of the work in the literature focuses on the study of physical measurable variables such as the amount of pollen in the air, vegetative indices that indicate plant health, or rainfall maps. However, this prediction is considered, since these variables are captured in springtime between April and May, when there are already other visible features that allow agricultural technicians to predict how abundant the next harvest will be. On the contrary, an early prediction cannot make use of these directly observable variables, but uses the historical behavior of the place (at the municipality level) and the environmental and climatic variables of the immediately preceding months. This leads us to predict the behavior of the next harvest

approximately because not all the factors that will affect are known at this stage. However, it is possible to advance what the general trend will be. In fact, some other factors are decisive during the following months: the processes of flower pollination, the fruit setting and its subsequent ripening. However, the behavioral trend of the next crop can be obtained with controlled error with the regression techniques of our model. In any case, this predictive model will be fed as the seasons progress to better adjust to the time of harvest."

Reviewer comment: 2.2 In line 178, a typo: 'stydy'

Author response: Solved. Thank you very much.

Reviewer comment: 2.3 Creating a table for three numbers is unnecessary (Table 1)

Author response: That's right, the table has been removed

Reviewer comment:3. Data and method
3.1 In line 346, 'in which only those plots of olive trees have been considered', Does this imply that several cities (municipalities) are selected from all 97 municipalities here? Please provide clarification and mapping details.

Author response: There were selected all 97 municipalities, each one is made up of several olive groves. To clarify this in the text, this sentence has been added in the Introduction section.

"Note that each municipality is made up of several cities and towns".

Reviewer comment: 3.2 In line 347, it mentioned 'process the spectral images', what kind of information has been collected from Google Earth Engine? The polygons in each city or others?

Author response: Google Earth Engine has been used for the calculation and analysis of vegetation indices from Sentinel-2 imagery.

Reviewer comment:3.3 In line 349, what is simplification process? More details should be provided regarding this geoprocessing (remove line strings in a defined polygon in Figure 2 ?).

Author response: This comment has been considered and the following text has been added to the last paragraph of the 3.1 section:

"This involves generalizing the polygons that define the outline of each municipality to simplify their geometry as much as possible in order to lighten the volume of the shapefile"

Nevertheless, following the indications of editor, this figure has been delete in the last version because the Creative commons share-alike 3.0 license used for figure 2 is not compliant with the licensing rules of the journal.

Reviewer comment: 3.4 For line 360 to 372, why is the olive growing cycle discussed in the dataset instead of incorporating relevant literature to establish a connection with the study's objective, such as early and late prediction?

Author response: This fact is important because we select specific variables in our study depending on the phenology of the olive plant, and we connect these processes with the seasons we divide the dataset acquisition to feed the ML algorithm.

On the other hand, to the best of our knowledge, early prediction in the literature regarding olive crops is almost nonexistent. We have highlighted this issue in the text, including a new figure (Figure 1). Therefore, literature review is focused on the set of variables used in similar studies and the machine learning processes as well.

Reviewer comment: 3.5 In subsection 3.2.2 Weather information, why is the weather variable, sourced from satellite data, treated separately from the satellite data discussed in section 3.2.3 ? Any specific intention? Furthermore, please explain how the three labels mentioned from line 435 to 443 are derived based on temperature and precipitation. Another concern is the presentation of Temperature and Rainfall data in 3.2.3, which requires clarification (different information or other intentions).

Author response: The reason is because it was originally considered to use meteorological information from ground-based weather stations. And in fact it was proven to be good for the predictive model. However, given the scale at which we work, it was justified to better use satellite images. Secondly, This section has been clarified by changing the tables to Figures 5 and 6 (Figures 4 and 5 in the last version of the manuscript) where the analysis performed to select the best set of predictor variables for the model is justified.

Reviewer comment: 3.6 In section 3.2.3 satellite data, the focus should be more on the indices utilized rather than on not using them in this study. Therefore, consolidating all indices and their respective data sources into one table can enhance clarity in this section.
Author response: Yes, it's right. We have deleted the table and we only comment on which of these indices are really important for our study. We only mention the set of unselected indices in a couple of sentences.

Reviewer comment: 3.7 In table 6, the MSI is not included in Table 5.

Author response: Yes, thankyou, the index has been included now.

Reviewer comment: 3.8 In line 528, what is ECMWF (have not found any clues through the whole manuscript)?

Author response: This comment has been considered and the following aclaration has been added to the text:

“This is the European Centre for Medium-Range Weather Forecasts, is an independent intergovernmental organization supported by many European nations. It specializes in producing and disseminating numerical weather predictions, which are crucial for meteorological services worldwide”

4. Model

Reviewer comment: 4.1 In Initial data preparation, any results or any presentation for each distribution of the variable (in result section)? Or can a statistical description of all independent variables and dependent variables be provided in a Table?

Author response: This suggestion has been considered and in order to clarified this issue Figure 5 and 6 (this numbering corresponds to the latest version of the manuscript) have been added. This figures shows the different results obtained in the prediction models using different subsets of independent variables.

Reviewer comment: 4.2 In line 567, what kind of algorithm was used in anomaly identification?

Author response: In this “Work flow process” section it is explained how the SVM algorithm is used in order to identify anomalies in the dataset.

Reviewer comment: 4.3 In data transformation, does it refer to the standardization or normalization of the data? If so, what specific technique is being referred to? Please provide the equation for the technique mentioned.

Author response: This suggestion has been considered and the following text has been added:

“As an example, the specific transformations that have been made to the data are those that are typical of multi-source data integration. In the case of the meteorological data, file format conversions have been necessary, and in the case of the vegetation

indices, a classification and subsequent labelling of the ranges has been required in order to be able to insert them into the models."

Reviewer comment: 4.4 In line 581, what is the 'unify all these data into a single source'?

Author response: It means integrating multi-source data so that it can be part of the input dataset in the model.

Reviewer comment: 4.5 Does the point 7 mean the feature selection? What is the meaning of 'we also check different ways of measuring error'? using different indices?

Author response: In order to measure error we use two different metrics: Mean Absolute Error (MAE) and also Root Mean Squared Error (RMSE). In order to clarify this issue in the text in section 3.3.2 has been added the following text:

"The RMSE, Equation 2, is also calculated for each model, because the two metrics are complementary. MAE measures average absolute errors, offering robustness against outliers and straightforward interpretability. However RMSE emphasizes larger errors due to its squaring step, making it sensitive to outliers and suitable for scenarios where large errors are critically undesirable. RMSE can disproportionately affect model evaluation due to its emphasis on larger errors".

Reviewer comment: 4.6 The 8 points can be aggregated to workflow process subsection by providing more details of model/algorithms used in each step. Section 3.3 can be organized flowing the workflow processing orders.

Author response: This suggestion has been taken into consideration and in order to clarify this issue the following text has been added the "Workflow process" section

"As described in the State of the Art section, the type of target in this study makes regression algorithms the ideal ones to perform the regression, however, it is initially unknown which one will work best. To select the best one, it is necessary to perform preliminary tests and analyze the errors of each one. In this case, the preliminary tests completely ruled out the use of the GLM, since the resulting errors were not tolerable. The reason for such results is that it is a pure linear algorithm, prevailing nonlinear relationships between the variables of the dataset. Random Forest, SVM (with linear and Gaussian kernel) and neural networks were then applied. Although in their basic forms some of these algorithms inherently handle linear separations (such as linear SVM), all three can be extended or inherently possess the ability to model nonlinear relationships".

Reviewer comment: 4.7 From line 609 to 620, please clarify how SVM has been applied to AD. If so, is it employed for classification, and if it is, is it binary or multi-class classification? Additionally, which type of outliers will be eliminated in this

context? Alternatively, is this technique referring to unsupervised one-class SVM? Another concern is the intention of SVM used for AD in this dataset? It also indicates that the performance of GLM is worse compared to SVM; to what degree is this disparity?

Author response: SVM is used in the pre-detection of anomalies in the data. In this case, a complexity criterion is set from which the algorithm does not consider in the calculations those data that exceed a threshold. From there, to generate the prediction, the SVM performs an unsupervised prediction of non-categorical numerical values.

Reviewer comment: 4.8 What kind of index (why the value of feature above 1 is selected) is used and how the MDL help to select the features? In Line 630, it mentioned model selection which need more specification.

Author response: The MDL algorithm is mentioned because in the case of initial variable selection it is very helpful and serves to guide the reader, specially when we have a large set of variables. However, no further details are given in the article because the results of the algorithm coincided with the selection made by the olive crop expert and the number of variables was not too large.

Reviewer comment: 4.9 What is the meaning of 'the restrictions only depend on the limitations imposed by the hardware used' in line 659?

Author response: Refers to the computational capacity of the computer.

Reviewer comment: 4.10 In line 671, what is the intention of categorization? Does this mean that it transitions from a regression task to a classification task?

Author response: Absolutely not, it refers to the label of Vegetation Index Maps to insert the values in the regression model in order to avoid the overfitting.

Reviewer comment: 4.11 From line 680 to 683, it should be more specific while you use the MAE, the n is the sample numbers, so if the prediction for one year is based on 97 cities (the geospatial unit of analysis should also be mentioned in the study area), so the n is 97. If you implement 10 times prediction for 10 years, you will get 10 MAEs here.

Author response: Certainly in the MAE equation "n" is the number of the sample, this is the number of municipalities. Therefore, in the cross validation an MAE is calculated for each year, however to make the comparison between different algorithms, the average MAE for all years is taken as the reference value, that is why only one value is shown.

Reviewer comment: 4.12 In line 690, what is the value of k?

Author response: The k-fold cross-validation technique is a statistical method used to estimate the skill of machine learning models.

Reviewer comment: 4.13 The hyperparameter processing SVM is missing, i.e., selection the optimised parameters?

Author response: It was used the default parameters used by the system (Oracle Data Mining). They are:

Standard Deviation 2.028.306 Complexity Factor 0.589921 Kernel Function Gausiano Algorithm Name Support Vector Machine Active Optimisation Enable Automatic Preparation Enable SVMS_EPSILON 0.040708 Core Cache Size 50,000.000 Tolerance 0.001

Reviewer comment: 4.14 The olive oil is litres in Figure 3 but Kg in other places in the manuscript.RMSE

Author response: This comment has been taken into consideration and the text in the Figure has been corrected. “Litres” has been replaced by “Kg”.

Reviewer comment: 4.15 A concern is that what is the mathematic representation of VI index for a city-level as an input in the SVM model? A vector or a matrix? Please make more specific for each variable input and the sample numbers (i.e., cities and annual months) in the beginning of the workflow introduction.

Author response: Each municipality is assigned the average value of each VI. The average of the pixel values found within the polygon that covers each municipality is averaged. This operation is performed using the cloud-based platform Google Earth Engine.

5. Results and discussion

Reviewer comment: 5.1 It is preferable to create a plot depicting the points based on predicted values and actual values. This approach facilitates the generation of metrics such as R-square (RMSE and MSE can be considered as well). Furthermore, why MAE is solely showed in the training set and not the testing set as it mentioned trained using data from seven years to predict outcomes for another year in line 697. Another concern of MAE is the type of value utilized for calculation, whether it's the

normalized value (ranging from -1 to 1) or the actual value (potentially 1000,000 Kg of olive crop). Demonstrating the error in actual values is essential for assessing the efficiency and accuracy of the SVM in predicting the olive crop and oil.

Author response: The authors are grateful for the comment and to clarify the analysis of the accuracy of the prediction, figure 9 (figure 8 in the latest version of the manuscript) has been included in which the difference between the actual value and the prediction for a specific year is shown graphically for each municipality. Also, in the last paragraph of the results the following text has been added:

"As can be seen in the figure, in general, the SVM algorithm with Gaussian kernel models the prediction better than the rest of the algorithms. However, when working on a larger scale, with a greater level of detail, the most appropriate algorithm for each municipality would have to be particularized since, as the detailed analysis shows, not all municipalities offer the same results"

Reviewer comment: 5.2 It is confused that line 715 involves predicting the year 2017 using data from 2018 to 2021. Could this be one part of the cross-validation procedure? There is a need for clarification regarding the training (including hyperparameters, cross-validation) and testing processes (alongside which part of data) involved in this case.

Author response: This comment is right, that refers to a part of the cross validation. Nevertheless, in order to clarify the explanation the following text has been added:

"In this sense, and in order to make a first selection of algorithms, initial tests were carried out using Random Forest, SVM (with linear and Gaussian kernel) and Neural Networks algorithms. When evaluating the year 2021, the training data covers the years 2009 to 2020 and 2022, deliberately excluding the 2021 data used to evaluate the effectiveness of the model. In this first phase of algorithm selection, a specific set of predictors, the meteorological variables, was used. In a later phase, once the most suitable algorithms had been selected, an exhaustive analysis was carried out to select the most suitable predictors for the prediction model. In this preliminary analysis, the RMSE obtained by each algorithm for the year 2021 was analyzed, Table ". Also, Table 6 has also added describing the parameters used in the testing process using the regression model.

Reviewer comment: 5.3 The discussion is brief, particularly regarding the limitations identified in this study, such as issues with the model and data representation.

Author response: The authors are grateful for the comment and to clarify the analysis of the accuracy of the prediction, figure 9 (figure 8 in the latest version of the manuscript) has been included in which the difference between the actual value and the prediction for a specific year is shown graphically for each municipality. Also, in the last paragraph of the results the following text has been added:

“As can be seen in the figure, in general, the SVM algorithm with Gaussian kernel models the prediction better than the rest of the algorithms. However, when working on a larger scale, with a greater level of detail, the most appropriate algorithm for each municipality would have to be particularized since, as the detailed analysis shows, not all municipalities offer the same results”

Reviewer 8#:

The aim of the study was to predict accurate yield data for olive groves in the province of Jaen in the Andalusia region of southern Spain at the beginning of the growing season using machine learning algorithms and a thorough analysis of the predictor variables.
The outline of the paper is generally appropriate and has some strengths, although some major details need to be revised before acceptance:

Reviewer comment: 1. The introduction (section 1) lacks proper referencing, making it essential to include references in the sentences to support the content effectively.

Author response: We have rewritten most paragraphs of this section. Most of the important concepts of the section are expanded in Section 2, adding the associated references.

Reviewer comment: 2. The second part deals well with the subject of theory and also the review of related studies, in this sense this part was well written.

Author response: Ok, thank you.

Reviewer comment: 3. The third section of the article elucidates the study area, providing comprehensive details on data collection and corresponding analysis, presented in a well-crafted manner.

Author response: Ok, thank you.

Reviewer comment: 4. The section on the results is also acceptable, as it clearly presents the results in two subsections - prediction based on meteorological variables and prediction based on the vegetation index.

Author response: Ok, thank you.

Reviewer comment:5. Nevertheless, it is essential to present the discussion section clearly in order to enable a thorough examination of the results and their comparison

with the findings from other studies. In this way, the unique contributions of the authors in the context of this study should be emphasised.

Author response: The authors are grateful for the comment and to clarify the analysis of the accuracy of the prediction, figure 9 (figure 8 in the latest version of the manuscript) has been included in which the difference between the actual value and the prediction for a specific year is shown graphically for each municipality. Also, in the last paragraph of the results the following text has been added:

“As can be seen in the figure, in general, the SVM algorithm with Gaussian kernel models the prediction better than the rest of the algorithms. However, when working on a larger scale, with a greater level of detail, the most appropriate algorithm for each municipality would have to be particularized since, as the detailed analysis shows, not all municipalities offer the same results”

Reviewer comment: 6. In addition, as can be seen in the conclusion section, the strengthening of the content in relation to the research implication as well as its limitations and suggestions for future studies should be clearly presented.

Author response: This suggestion has been considered and the Conclusions section has been revised. The following paragraph has been added:

“In this research, predictive models of crop yield at an early stage of the olive growing season in Spain has been generated. This is before spring, just when the previous year's harvesting season has just ended and there is still no evidence in the field of how productive the next season will be. The targets of the predictive models are both olive fruit yield and oil yield. These are continuous numerical data, hence regression algorithms have been used when applying ML. It has been confirmed the SVM algorithm with Gaussian kernel provides the best predictive accuracy”.

Reviewer 9#:

The authors have addressed all the questions and comments, and this manuscript is good to publish. The statistical analysis has been performed well according to the data obtained. The English Language and grammar meet the requirements to get published in Plos One. This research work will be helpful for better understanding of the early season crop yield prediction with machine learning as it is demanding in the industry and its a new approach in this field.

Reviewer 10#:

Reviewer comment: The study may focus on the machine learning section
In general, the article should be reviewed in accordance with the journal writing rules.
table 6 needs to be reorganized
Results section should be expanded.

Author response: These comments have been carried out and the Results section has been revised. The changes are indicated in red color en el text. also new figures have been added.

Reviewer 11#:

I appreciate the authors' effort in applying SVM to predict crop yields using temporal data. However, there are several limitations and areas of improvement that I'd like to highlight:

Reviewer comment:1. A more detailed comparison with traditional time series analysis methods is necessary. It would be beneficial to include a discussion on the specific advantages of SVM in handling the nonlinearities and complexities of the data involved in this study.

Author response: This comment has been taken into account and the following text has been added the “Workflow process” section

“As described in the State of the Art section, the type of target in this study makes regression algorithms the ideal ones to perform the regression, however, it is initially unknown which one will work best. To select the best one, it is necessary to perform preliminary tests and analyze the errors of each one. In this case, the preliminary tests completely ruled out the use of the GLM, since the resulting errors were not tolerable. The reason for such results is that it is a pure linear algorithm, prevailing nonlinear relationships between the variables of the dataset. Random Forest, SVM (with linear and Gaussian kernel) and neural networks were then applied. Although in their basic forms some of these algorithms inherently handle linear separations (such as linear SVM), all three can be extended or inherently possess the ability to model nonlinear relationships”.

Reviewer comment 2. The results section primarily relies on point estimates for performance metrics. In real-world scenarios, understanding the uncertainty around these estimates is crucial. The authors should consider providing confidence intervals.

Author response: The authors agree with the reviewer that the analysis of the results in their current state does not clearly reflect the accuracy of the models generated. For this reason, Figure 9 (Figure 8 in the latest version of the manuscript) in which the difference between the actual value and the prediction for a specific year is shown graphically for each municipality. Also, in the last paragraph of the results the following text has been added:

“As can be seen in the figure, in general, the SVM algorithm with Gaussian kernel models the prediction better than the rest of the algorithms. However, when working on a larger scale, with a greater level of detail, the most appropriate algorithm for each municipality would have to be particularized since, as the detailed analysis shows, not all municipalities offer the same results”

Reviewer comment 3. For any machine learning study, it's crucial to compare the performance against standard baselines.

Author response: This comment has been considered and regression algorithms has been performed comparing them with a pure linear regression as it is GLM algorithm. The following text has been added in the “Modelling methodology” section:

“The choice of crop prediction algorithms depends mainly on the ability to handle large and complex data sets with multiple input variables, which is our case. It also depends on the need to model linear or non-linear relationships and whether the types of data to be predicted are continuous or categorical. In addition, algorithms are required that are able to capture the interactions between the various agricultural factors that affect the yield of the olive crop, such as yield data from previous years, weather conditions and the spectral response of the crop as a function of soil properties, plant condition and agricultural practices. Our variable to be predicted, the crop yield, is a numerical and continuous data, so regression algorithms were applied, and within these, Random Forest, GLM, SVM with linear kernel and Gaussian were selected, and finally, tests with Neural Networks were also carried out. The reasons are that Random Forest, GLM, SVM with linear and Gaussian kernels, and Neural Networks share the ability to model complex nonlinear relationships through specific structures and transformations. Unlike regression algorithms such as Gradient Boosting Machines, which sequentially correct prior errors, or K-Nearest Neighbors, which depends on data proximity, these models can address a greater diversity of patterns in the data. In addition, they differ from Deep Learning techniques, which use multiple layers to extract progressively more abstract features and are more suitable for large volumes of data and unstructured features such as images and text”.

And also, in the “Workflow process” section:

“SVM is based on the fundamental idea of minimizing the hypersphere of the single class of examples in the training data, and consider all other examples outside the hypersphere as outliers or outside the distribution of the training data. This algorithm generates a prediction and a probability for each case in the score data. If the prediction is 1, the case is considered typical. If the prediction is 0, the case is considered anomalous. This behavior reflects the fact that the model is trained on data following a normal distribution. Also Generalized Linear Models (GLM) which is a pure linear regression algorithm is used, but it has been discarded because in early tests it performed worse than SVM”.

Reviewer comment 4. This work doesn't thoroughly justify the choice of the SVM model for this particular task. Are there specific characteristics of the dataset or problem domain that make SVM particularly suited, or was it an arbitrary choice?

Author response: The choice of SVM was not arbitrary; a cross validation was carried out for all years of the MAE, obtaining better values for this algorithm compared to GLM, Random Forest and even neural networks. The RMSE was also analyzed, confirming this selection.

Reviewer comment 5. SVM has several hyperparameters, such as the regularization constant (C), the kernel type (linear, polynomial, radial basis function, etc.), and parameters specific to those kernels (like degree for polynomial or gamma for RBF). These parameters can significantly affect performance. Not discussing them leaves out crucial aspects of the model's behavior. Also, without knowledge of the chosen hyperparameters, there's the concern of potential overfitting. For example, an SVM with a small value of C might fit the training data very closely, possibly capturing noise and leading to poor generalization on unseen data. To strengthen the paper, the authors should have included details on the SVM parameters, the rationale behind their selection, and potentially even results from a hyperparameter tuning process. This would provide more clarity and confidence in the presented results.

Author response: This comment has been taken into account and Table 6 with the chosen hyperparameters has been added. Also the following comment:

“In this sense the default parameters were used by the system, Oracle Data Mining”

Reviewer 12#:

Reviewer comment 1-The abstract must clearly articulate the originality of the investigation.

Author response: This suggestion has been considered and the abstract has been rewritten. The changes made to the text are as follows (added in red)

In the production sector, the usefulness of predictive systems as a tool for management and decision-making is well known. In the agricultural sector, a correct economic balance of the farm depends on making the right decisions. To this end, having information on crop yields for the next harvest when the current crop has just been harvested is an extraordinary help. At that time, there is no visible indication in the field of how productive or unproductive the next harvest will be. Numerous predictive models infer accurate crop yield data from aerobiological variables and pollen analysis; this is around spring, in the middle stage of the crop year, when planning and investments are already done. The aim of this study is to anticipate accurate crop yield data at an early stage of the cropping season. In the case of olive groves in Spain, this period is in February. This work is developed for an entire province, Jaen, belonging to the region of Andalusia, in Southern Spain. The methodology uses Machine Learning algorithms together with an exhaustive analysis of predictor variables. Temporal data come from public web services, such as spatial data infrastructures of some state agencies. The processing of the satellite imagery is carried out by the geospatial processing service Google Earth Engine. The result is the early prediction of kilograms of both olive fruit ~~crop~~ and olive oil crop, eight months prior to the beginning of the first harvesting

campaigns of the year, with an average absolute error of prediction better than 26%. The relevance of this work is the early availability of predicted crop yield data together with the multi-scale applicability of the predictive models. This makes this model a useful tool for all the agents involved in olive grove management. From farmers, to agricultural technicians, researchers and scientists dedicated to the study of the olive tree, to governmental institutions and agricultural associations that provide technical support, advice and regulation to ensure responsible practices and the long-term viability of the olive industry.

Reviewer comment 2-The authors did not specify the method used to determine the sample size.

Author response: In order to validate the accuracy of the models it is applied the k-fold cross-validation technique . This approach entails evaluating the model's predictive capability by isolating the data from a particular year and segregating it from the data used for model training. In the context of this research, it is utilized to validate the models' accuracy in predicting crop and oil production for a specific year. In the cross-validation process, the prediction for each year is checked.

Reviewer comment 3-If the authors draw A flowchart to the workflow in the methodology, the part will help link the audiences to how this method advances current knowledge and will help the audiences follow the results quickly.

Author response: This comment has been considered and figure 1 has been replaced in order to clarify the actual knowledge and our contribution.

Reviewer comment 4-The researchers need to mention the experimental design they used in this manuscript to help the readers follow the results and the conclusions.

Author response: This comment has been considered and in the Introduction section has been revised de following text in order to clarify the experimental design of our research:

“In olive growing, early prediction is considered to be that which precedes pollination, that is, in the months of February and March (in the northern hemisphere). In the particular case of the olive grove, most of the work in the literature focuses on the study of physical measurable variables such as the amount of pollen in the air, vegetative indices that indicate plant health, or rainfall maps. However, this prediction is considered \textit{late prediction}, since these variables are captured in springtime between April and May, when there are already other visible features that allow agricultural technicians to predict how abundant the next harvest will be. On the contrary, an early prediction cannot make use of these directly observable variables, but uses the historical behavior of the place (at the municipality level) and the environmental and climatic variables of the immediately preceding months. This leads us to predict the behavior of the next harvest approximately because not all the factors that will affect are known at this stage. However, it is possible to advance what the general trend will be. In fact, some other factors are decisive during the following months: the processes of flower pollination, the fruit setting and its subsequent ripening. However, the behavioral trend of the next crop

can be obtained with controlled error with the regression techniques of our model. In any case, this predictive model will be fed as the seasons progress to better adjust to the time of harvest. "

Reviewer 13#:

Reviewer comment 1. Based on the detailed examination of the "Materials and Methods" section and subsequent results and discussion, the manuscript demonstrates a methodologically sound approach to predicting olive crop yields using satellite imagery and machine learning techniques, specifically SVM with Linear and Gaussian kernels. The study incorporates rigorous data preprocessing, anomaly detection, and transformation steps to optimize the input for predictive modeling. It also employs regression and analysis and evaluates the influence of various predictors on the target variables (kg of live fruit and olive oil).
The use of k-fold cross-validation for model accuracy assessment and the detailed analysis of mean absolute error (MAE) across different models and predictor sets further illustrate the rigorous statistical and experimental methods applied. The results section substantiates the effectiveness of integrating meteorological variables and vegetation indices into the predictive model, highlighting improvements in prediction accuracy through these methods.
Given these observations the manuscript can be considered partly technically sound as it follows rigorous experimental procedures, employs appropriate controls (e.g., cross-validation), and uses relevant sample sizes for the machine learning model development and validation process. The conclusions drawn from the data presented are supported by the analysis conducted, indicating that the study's outcomes are rooted in the data obtained and processed through the described methodologies.

However, the assessment of "partly" also acknowledges that while the manuscript demonstrates technical rigor in its approach, the effectiveness and robustness of the predictive models could benefit from further validation across broader datasets or independent validation sets to strengthen the conclusions drawn. Additionally, discussions on the limitations of the study, potential biases in the data or model, and the generalizability of the findings to other regions or crop types would enhance the manuscript's depth and scientific robustness.

Author response: This comment has been considered and RMSE of each model has been added in order to compare the precision of each algorithm. Also, figure 9 (figure 8 in the latest version of the manuscript) has been included in which the difference between the actual value and the prediction for a specific year is shown graphically for each municipality. Also, in the last paragraph of the results the following text has been added:

"As can be seen in the figure, in general, the SVM algorithm with Gaussian kernel models the prediction better than the rest of the algorithms. However, when working on a larger scale, with a greater level of detail, the most appropriate algorithm for each municipality would have to be particularized since, as the detailed analysis shows, not all municipalities offer the same results"

Reviewer comment 2. Based on the review of the "Materials and Methods" section and related content, including the statistical methods applied for data analysis and model evaluation, the statistical analysis in the manuscript has been performed appropriately and rigorously.

Author response: The authors are grateful for this comment

Reviewer comment 3. Based on the information provided in the “Materials and Methods” and other relevant sections of the manuscript, the authors have indicated that the primary data sources for their study are publicly accessible, including datasets from regional governmental institutions and publicly available web servers for meteorological data. The use of Google Earth Engine for processing satellite images is also mentioned, suggesting the data used in the study are accessible through public platforms or repositories. However, the manuscript does not provide explicit details on accessing these datasets, such as direct links, access instructions, or deposit information in a public repository specific to the study’s datasets.
Given the lack of explicit instructions or links for direct data access or the deposit of processed data in a recognized public repository, and in the absence of a clearly stated Data Availability Statement that meets the PLOS Data policy’s requirements for unrestricted access to all underlying data, the answer would be “No”. The manuscript does not fully comply with the requirements to make all data underlying the findings fully available without restrictions, as per the PLOS Data policy guidelines.

Author response: All data used in this paper are satellite free access data, specifically MODIS and LandSat 7 and 8 satellites. Although, the access and the processing of such data has always been done through Google Earth Engine, which facilitates these processes without downloading the information locally and by means of scripting. Results can be downloaded as CSV information and/or RGB images. This numerical information is the input to our model. We have indicated this in the text and also included a new image, Figure 4 (Figure 3 in the latest version of the manuscript), as a descriptive map in which we can visualize the NDVI index along the province of Jaen.

Reviewer comment 4. Based on the sections of the manuscript reviewed, including the abstract, introduction, materials and methods, results and conclusions, the manuscript is presented in an intelligible fashion and is written in standard English. The test maintains a formal and scientific tone, appropriate for an academic audience, with technical terms and concepts explained in a manner that should be accessible to readers familiar with the field. There are no significant typographical or grammatical errors noted in the sections reviewed, and the manuscript follows a logical structure that facilitates understanding of the study’s objectives, methods, results and implications.

Author response: The authors are grateful for this comment
